# Exercise modalities and dose-response for LVEF Improvement in heart failure patients: a systematic review and network meta-analysis

**DOI:** 10.3389/fcvm.2026.1834798

**Published:** 2026-06-05

**Authors:** Fengrui Shi, Xiangao Li, Hong Wang

**Affiliations:** 1 Wuhan Sports University, Wuhan, China; 2 Hainan University, Haikou, China

**Keywords:** cardiac rehabilitation, exercise dose, heart failure, left ventricular ejection fraction, network meta-analysis

## Abstract

**Objective:**

To compare the effects of various exercise modalities and doses on left ventricular ejection fraction (LVEF) in patients with heart failure.

**Methods:**

We systematically searched eight electronic databases through December 2025. Randomized controlled trials involving adult HF patients were eligible. Interventions comprised aerobic exercise (AE), resistance training (RT), combined exercise (CE), mind-body exercise (ME), high-intensity interval training (HIIT), and control. Random-effects network meta-analysis was employed to estimate mean differences with 95% credible intervals, and surface under the cumulative ranking curve probabilities were calculated. Nonlinear dose-response relationships were modeled using MET-minutes per week.

**Results:**

42 RCTs comprising 3,519 participants were included. Network meta-analysis demonstrated that all exercise modalities significantly improved LVEF compared with control. Resistance training showed the largest treatment effect (MD: 9.9; 95% CrI: 6.5, 13.0), followed by HIIT (MD: 8.4; 95% CrI: 4.2, 12.0), CE (MD: 6.0; 95% CrI: 2.9, 9.0), and AE (MD: 5.2; 95% CrI: 3.3, 7.1). SUCRA rankings indicated RT had the highest probability of being optimal (93.1%), followed by HIIT (76.1%), ME (55.3%), CE (46.0%), and AE (29.5%). Dose-response analysis revealed a non-linear quadratic relationship between MET-minutes per week and LVEF improvement. The minimum effective dose was 280 MET-minutes/week, with the optimal dosage range of 500–800 MET-minutes/week. Modality-specific patterns emerged: RT produced benefits at low doses (220 MET-minutes/week; optimal 330–440), whereas HIIT required higher thresholds (440 MET-minutes/week; optimal 560–780). Evidence certainty ranged from very low to moderate across comparisons per CINeMA framework.

**Conclusions:**

Resistance training is the most effective exercise modality for improving LVEF in HF patients, particularly at low-to-moderate doses. These findings support a precision-based rehabilitation strategy centered on RT to optimize cardiac function.

**Systematic Review Registration:**

PROSPERO, CRD420261329239.

## 1 Introduction

Heart failure represents a substantial global public health challenge with escalating prevalence and considerable economic burden on healthcare systems (1). As the terminal stage of various cardiovascular diseases, HF is characterized by structural and functional cardiac abnormalities impairing ventricular filling or ejection capacity (2). Left ventricular ejection fraction (LVEF) serves as a fundamental parameter for assessing cardiac systolic function, HF severity, and patient prognosis (3). Despite significant advances in pharmacological therapies, prognosis remains suboptimal, highlighting the need for effective non-pharmacological interventions to improve cardiac function.

Exercise rehabilitation has been recommended by multiple clinical guidelines as an integral component of standard care for HF patients (4). Numerous randomized controlled trials and meta-analyses have demonstrated that regular exercise training significantly improves exercise tolerance, quality of life, and clinical prognosis (5). A recent systematic review and meta-analysis by Gupta et al. (2024) specifically examined the effects of physical exercise on LVEF, health-related quality of life, and other cardiorespiratory parameters in cardiac patients, reporting significant improvements in LVEF (pooled effect size: 0.45; 95% CI: 0.32–0.58), peak VO₂, and quality of life across diverse exercise modalities (6).Various modalities are applied in HF rehabilitation, including aerobic exercise (AE), resistance training (RT), high-intensity interval training (HIIT), combined exercise (CE), and mind-body exercise (ME) (7). However, the relative impact of different exercise modalities on LVEF—a pivotal indicator of cardiac function and their comparative efficacy remain contentious (8).

The relationship between exercise dose and cardiac benefits has garnered increasing attention. Dose parameters may exert heterogeneous effects on LVEF improvement across modalities. However, previous studies have not systematically integrated dose parameters or delineated optimal dose combinations for maximizing LVEF benefits (9). Conventional pairwise meta-analyses can only compare two interventions directly, precluding simultaneous evaluation of multiple modalities. Incorporating dose-response meta-analysis allows delineation of continuous relationships between exercise dose and outcomes, identifying minimum effective and optimal doses. This study aims to evaluate the effects of different exercise modalities on LVEF through systematic review and network meta-analysis, and to explore dose-response relationships, providing high-level evidence for precision exercise prescription.

## 2 Methods

This systematic review was conducted according to PRISMA-NMA guidelines (Supplementary Appendix 1) and registered with PROSPERO (CRD420261329239).

### 2.1 Search strategy

Due to the language proficiency of the research team and resource constraints, only studies published in English or Chinese were included. These two languages represent the primary publication languages in biomedical research and encompass the vast majority of high-quality cardiovascular rehabilitation trials. Nevertheless, this restriction may introduce language bias by excluding relevant studies published in other languages, a limitation that is further addressed in the Discussion section. We systematically searched PubMed, Embase, Cochrane Library, Web of Science, China National Knowledge Infrastructure (CNKI), Wanfang Database, Weipu Database, and Chinese Biomedical Database (CBM) databases from inception to December 2025. Search terms included “heart failure,” “exercise,” “left ventricular function,” and “randomized controlled trials.” The complete PubMed search strategy is provided below as an exemplar:

#1 “Heart Failure”[MeSH]
#2 “Cardiac Failure”[Title/Abstract] OR “Congestive Heart Failure”[Title/Abstract] OR “Heart Decompensation”[Title/Abstract] OR “Heart Failure, Congestive”[Title/Abstract] OR “Heart Failure, Left-Sided”[Title/Abstract] OR “Heart Failure, Right-Sided”[Title/Abstract] OR “Left-Sided Heart Failure”[Title/Abstract] OR “Myocardial Failure”[Title/Abstract] OR “Right-Sided Heart Failure”[Title/Abstract]
#3 #1 OR #2
#4 “Exercise”[MeSH] OR “Physical Exertion”[MeSH] OR “Exercise Therapy”[MeSH] OR “Resistance Training”[MeSH] OR “High-Intensity Interval Training”[MeSH] OR “exercise”[Title/Abstract] OR “physical activity”[Title/Abstract] OR “aerobic training”[Title/Abstract] OR “aerobic exercise”[Title/Abstract] OR “endurance training”[Title/Abstract] OR “endurance exercise”[Title/Abstract] OR “resistance training”[Title/Abstract] OR “resistance exercise”[Title/Abstract] OR “strength training”[Title/Abstract] OR “strength exercise”[Title/Abstract] OR “combined training”[Title/Abstract] OR “combined exercise”[Title/Abstract] OR “concurrent training”[Title/Abstract] OR “interval training”[Title/Abstract] OR “interval exercise”[Title/Abstract] OR “sprint training”[Title/Abstract]
#5 #3 AND #4
#6 “Ventricular Function, Left”[MeSH] OR “Stroke Volume”[MeSH] OR “Echocardiography”[MeSH] OR “ventricular function, left”[Title/Abstract] OR “left ventricular function”[Title/Abstract] OR “left ventricle function”[Title/Abstract] OR “LV function”[Title/Abstract] OR “cardiac function”[Title/Abstract] OR “heart function”[Title/Abstract] OR “systolic function”[Title/Abstract] OR “left ventricular systolic function”[Title/Abstract] OR “LV systolic function”[Title/Abstract] OR “ejection fraction”[Title/Abstract] OR “ejection fractions”[Title/Abstract] OR “fraction, ejection”[Title/Abstract] OR “fractions, ejection”[Title/Abstract] OR “ventricular ejection fraction”[Title/Abstract] OR “ejection fraction, ventricular”[Title/Abstract] OR “left ventricular ejection fraction”[Title/Abstract] OR “ejection fraction, left ventricular”[Title/Abstract] OR “LVEF”[Title/Abstract] OR “LV ejection fraction”[Title/Abstract] OR “left ventricle ejection fraction”[Title/Abstract] OR “ejection fraction, left ventricle”[Title/Abstract]
#7 #5 AND #6
#8 (“randomized controlled trial”[Publication Type] OR “randomized controlled trials as topic”[MeSH] OR “randomized”[Title/Abstract] OR “randomised”[Title/Abstract] OR “randomly”[Title/Abstract] OR “trial”[Title/Abstract] OR “placebo”[Title/Abstract])

Complete search strategies for each database are provided in Supplementary Appendix 2.

### 2.2 Study selection

We included RCT involving adult HF patients (≥ 18 years) in stable condition comparing structured exercise interventions versus control (usual care, placebo, or minimal lifestyle advice). Eligible exercise modalities comprised aerobic exercise (AE), resistance training (RT), high-intensity interval training (HIIT), combined aerobic and resistance exercise (CE), and mind-body exercise (BE), with intervention duration ≥ 2 weeks. The primary outcome was left ventricular ejection fraction (LVEF).

Exclusion criteria comprised non-randomized designs, case reports, trial protocols, animal studies, conference abstracts, reviews, studies with incomplete data or lacking baseline comparability, studies employing multiple combined modalities without distinct groups, and studies not reporting LVEF outcomes. Two reviewers independently screened titles/abstracts and full texts, with disagreements resolved by a third reviewer.

### 2.3 Data extraction

We extracted study design, sample size, baseline participant characteristics (age, BMI, health status), intervention parameters (modality, intensity, frequency, duration), and LVEF data (means and standard deviations of within-group changes from baseline to post-intervention).

### 2.4 Risk of bias and evidence assessment

Two reviewers (S.F.R. and L.X.G.) independently assessed the risk of bias of included studies using the Revised Cochrane Risk-of-Bias Tool for Randomized Trials (10). Five domains were evaluated: (1) randomization process, (2) deviations from intended interventions, (3) missing outcome data, (4) measurement of the outcome, and (5) selection of the reported result. An overall risk-of-bias judgment was subsequently assigned for each study. Risk of bias was categorized into three levels: low risk, some concerns, and high risk (11). Discrepancies were resolved through discussion with a third author (W.H.).

Evidence credibility was evaluated using the CINeMA framework across six domains: within-study bias, reporting bias, indirectness, imprecision, heterogeneity, and incoherence (12). The certainty of evidence was ultimately classified as high, moderate, low, or very low.

### 2.5 Physical activity dose computation and dose-response analysis

#### 2.5.1 Exercise intervention classification and dose standardization

Classification of exercise interventions followed the American College of Sports Medicine (ACSM) standards (13) and was performed independently by the two aforementioned reviewers.

Weekly exercise dose for each intervention group was standardized and expressed as metabolic equivalent of task-minutes per week (MET·min/week), calculated using the formula: Dose = Activity-specific MET value × Session duration (minutes) × Weekly frequency. For multi-component interventions, total dose was computed as the sum of constituent components. MET value assignment followed these rules: For studies explicitly reporting exercise speed or modality (e.g., walking 3.5–4.0 mph, cycling), corresponding MET values were obtained from the 2024 Adult Compendium of Physical Activities (14); and ACSM guidelines according to reported exercise intensity, modality, or %1RM (details in Supplementary Appendix 3). Missing data were requested from original authors via email.

#### 2.5.2 Network meta-analysis

To minimize baseline differences, change scores were calculated from pre- and post-intervention means and standard deviations. Bayesian random-effects network meta-analysis (NMA) was performed to estimate mean differences (MDs) with 95% credible intervals (CrI). Effect sizes were pooled using normal likelihood functions based on change-from-baseline values.

Heterogeneity was assessed using I^2^ statistics (> 50% indicating substantial heterogeneity) and expressed as the posterior median of *τ* with 95% CrI. Consistency was evaluated globally by comparing deviance information criterion (DIC) between consistency and unrelated mean effects (UME) models (ΔDIC < 5 indicating no significant inconsistency), and locally via node-splitting (*p* < 0.05 indicating inconsistency) (15). Treatment rankings were quantified using surface under the cumulative ranking curve (SUCRA) probabilities (0%–100%, higher values indicating greater probability of superior ranking). Forest plots and league tables were generated to present relative effects. Analyses were conducted in R using the gemtc package with Markov Chain Monte Carlo methods (4 chains, 20,000 iterations, 5,000 burn-in), with convergence assessed through trace plots (16).

#### 2.5.3 Dose-response network meta-analysis

Model-based network meta-analysis (MBNMA) with Bayesian random-effects models was employed to evaluate dose-response relationships between exercise dose and LVEF improvement. Key assumptions including network connectivity (17), consistency (18), and transitivity (19) were satisfied (Supplementary Figures S9-S11, Tables S9, S10). Multiple nonlinear models were fitted based on data distribution characteristics; after comprehensive comparison of model fit indices, the quadratic model was selected as optimal for characterizing nonlinear relationships (20). The minimum effective dose was estimated using 95% prediction intervals, and interventions were ranked based on improvement probability (21). In dose-response analyses, statistical significance was declared when the 95% CrI of effect estimates excluded the null value. All MBNMA and dose-response analyses were performed using the MBNMAdose package in R (version 4.5.1), with dose-response curves and visualizations generated using the ggplot2 package.

Network meta-regression was conducted to explore potential effect modifiers, including age, body mass index, sex ratio, intervention duration, frequency, and publication year. Sensitivity analyses were performed by sequentially excluding studies with high risk of bias and interventions with duration <4 weeks to assess result robustness.

## 3 Results

### 3.1 Study selection

**Figure 1:**
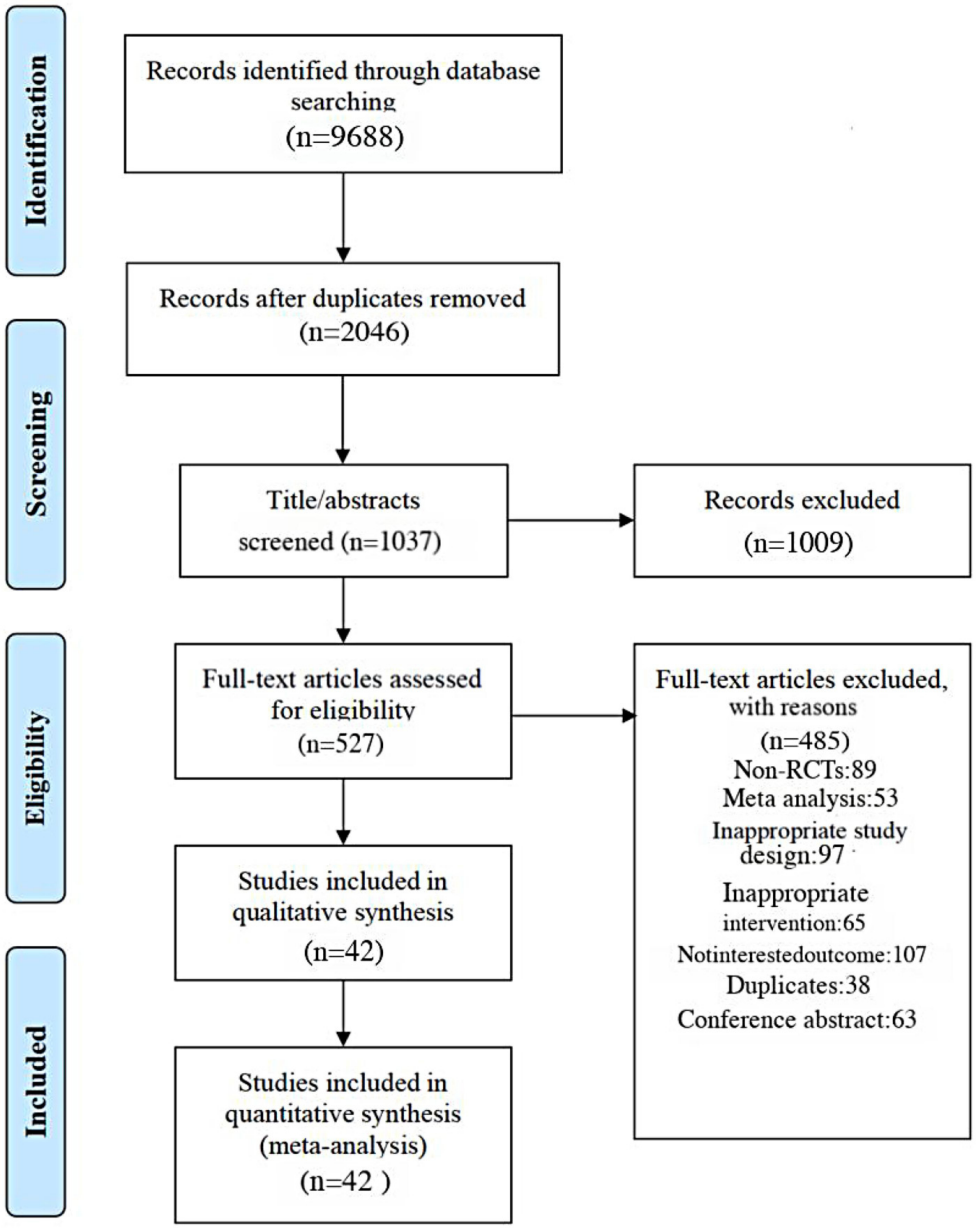
Flow diagram.

Systematic database searches identified 9,688 potentially relevant records. Following duplicate removal, 2,046 articles remained for title and abstract screening. Full-text review was conducted for all 527 articles meeting eligibility criteria for detailed assessment. Ultimately, 42 studies were included in the systematic review and meta-analyses. The complete screening and selection process is illustrated in Figure 1.

### 3.2 Characteristics of included studies

A total of 42 randomized controlled trials comprising 3,519 participants were included. The incorporated studies encompassed five exercise intervention categories: AE (22 studies), BE (8 studies), CE (6 studies), RT (5 studies), and HIIT (3 studies). Study distribution was markedly imbalanced across modalities, with AE representing 71% of all exercise intervention arms. This imbalance has important implications for network estimates and SUCRA rankings, as modalities with fewer contributing studies (particularly RT and HIIT) rely more heavily on indirect comparisons and have wider credible intervals. Detailed characteristics of included studies are provided in Supplementary Appendix 3.

### 3.3 Risk of bias and evidence assessment

**Figure 2:**
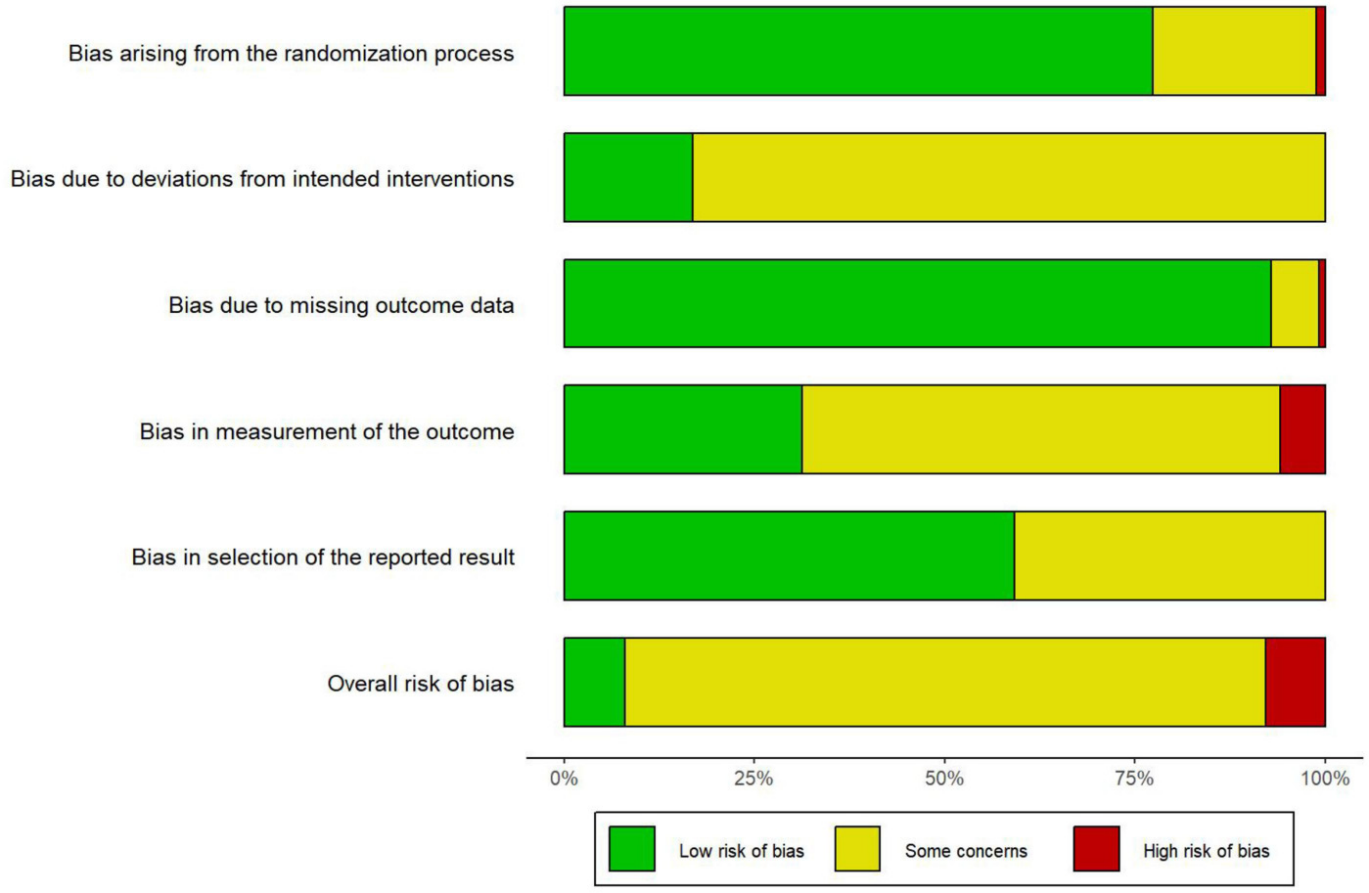
Risk of bias.

Among the 42 included studies, 5 were rated as high risk of bias, 34 as “some concerns,” and 3 as low risk of bias (Figure 2); domain-specific and overall risk-of-bias assessments for individual studies are provided in Supplementary Appendix 4. According to CINeMA evaluations of confidence in network comparisons, the certainty of evidence across comparisons ranged from “very low” to “moderate,” with detailed summaries presented in Supplementary Appendix 5.

### 3.4 Pairwise meta-analysis

Conventional pairwise meta-analyses demonstrated that all exercise interventions significantly improved LVEF compared with control (Supplementary Tables S2, S3). Combined exercise exhibited the largest effect size (MD: 5.86; 95% CrI: 4.46, 7.26; I^2^ = 62.5%), followed by aerobic exercise (MD: 8.51; 95% CrI: 6.80, 10.23; I^2^ = 23.1%), resistance training (MD: 6.70; 95% CrI: 4.40, 9.00；I^2^ = 0%), high-intensity interval training (MD: 5.07; 95% CrI: 1.93, 8.21；I^2^ = 0%), and mind-body exercise (MD: 4.89; 95% CrI: 3.66, 6.14; I^2^ = 33.2%). Significant heterogeneity was observed for the aerobic exercise versus control comparison (I^2^ = 62.5%), whereas heterogeneity for other comparisons was low to moderate. Publication bias assessment using funnel plot visualization and Egger's test revealed no significant asymmetry, suggesting absence of substantial small-study effects (Supplementary Appendix 6).

### 3.5 Network meta-analysis

#### 3.5.1 Model fit and consistency assessment

The random-effects consistency model demonstrated superior fit compared with the fixed-effects model (DIC: 121.6 vs. 309.0; residual deviance: 62.4 vs. 272.9). The between-study standard deviation was estimated at 3.19 (95% CrI: 2.36, 4.46), indicating moderate heterogeneity. Global consistency assessment comparing consistency and unrelated mean effects models showed similar DIC values (121.6 vs. 122.1), suggesting no significant inconsistency. Local inconsistency evaluation via node-splitting detected no significant discrepancies between direct and indirect evidence (all *P* > 0.05), confirming transitivity validity (Supplementary Table S5, Appendix 7).

#### 3.5.2 Relative treatment effects

Network meta-analysis revealed that all exercise modalities significantly improved LVEF compared with control (Figure 2). Resistance training demonstrated the largest treatment effect (MD: 9.93%; 95% CrI: 6.5, 13.0), followed by high-intensity interval training (MD: 8.37%; 95% CrI: 4.2, 12.0), combined exercise (MD: 5.99%; 95% CrI: 2.9, 9.0), and aerobic exercise (MD: 5.14%; 95% CrI: 3.3, 7.1).

#### 3.5.3 Treatment ranking

**Figure 3:**
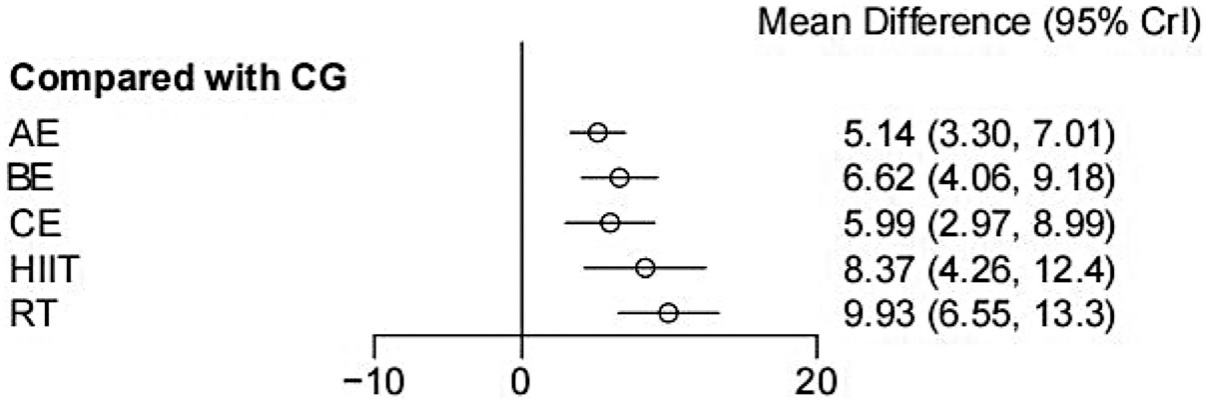
Forest plot for all studies.

SUCRA probabilities indicated that resistance training had the highest probability of being the optimal intervention (SUCRA = 93.1%), followed by HIIT (76.1%), mind-body exercise (55.3%), combined exercise (46.0%), and aerobic exercise (29.5%) (Table 1; Figure 3). Cumulative ranking probabilities consistently supported the superior therapeutic efficacy of resistance training.

**Table 1:** SUCRA table for all studies.

Treatment	(1)	(2)	(3)	(4)	(5)	(6)	SUCRA
AE	0	0.004	0.0755	0.3125	0.608	0	29.51
BE	0.026	0.179125	0.4375	0.246875	0.1105	0	55.27
CE	0.014	0.09925	0.300875	0.347	0.2385	0.000375	46.04
CG	0	0	0	0	0.000375	0.999625	0.01
HIIT	0.245375	0.48075	0.148875	0.083125	0.041875	0	76.09
RT	0.714625	0.236875	0.03725	0.0105	0.00075	0	93.08

### 3.6 Dose-response network meta-analysis

#### 3.6.1 Model selection and fit

Model-based network meta-analyses comparing different functional forms demonstrated that the quadratic model with random treatment effects provided the optimal fit to the data (DIC = 209.7, residual deviance = 62.3), significantly outperforming Emax, restricted cubic spline, and non-parametric models (Supplementary Table S7). Deviance plots confirmed adequate model fit, with the majority of data points contributing minimally to posterior mean deviance (Supplementary Figures S6, S7). Fitted plots revealed satisfactory agreement between predicted and observed values across all exercise modalities and dose levels (Supplementary Figures S8, S9).

Network connectivity was confirmed at both treatment and dose levels (Supplementary Figures S10, S11), and node-splitting analyses indicated no significant inconsistency for any specific dose comparison (all *p* > 0.05; Supplementary Table S8, Figure S12), supporting the transitivity assumption. Detailed summaries are provided in Supplementary Appendix 9.

#### 3.6.2 Overall dose-response relationship

**Figure 4:**
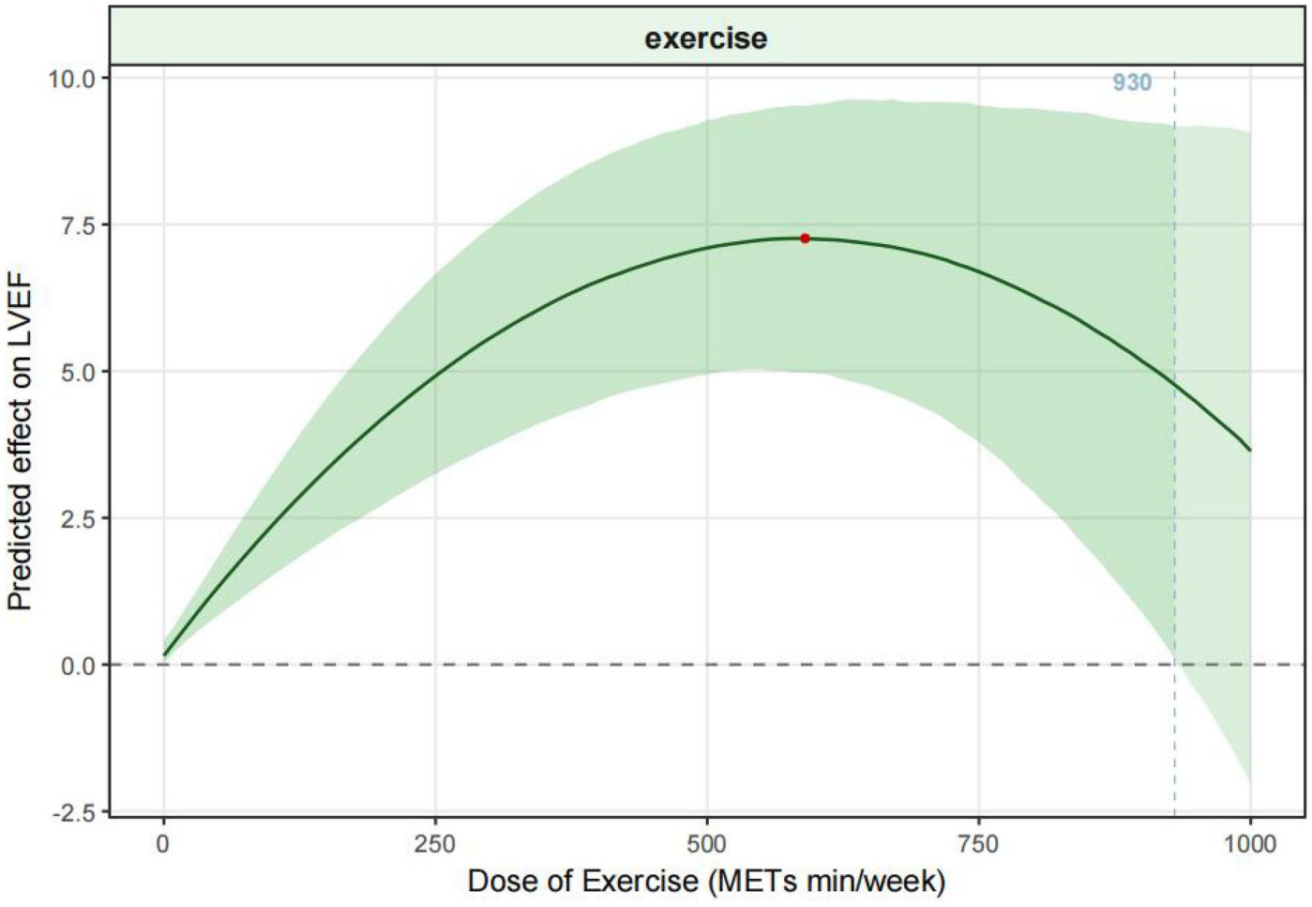
Dose-response relationship between total exercise volume and LVEF levels in patients with heart failure.the green area represents the 95% credible interval. MD indicates mean difference; METs, metabolic equivalents.

Dose-response analysis revealed a nonlinear relationship between weekly exercise dose (MET-minutes/week) and LVEF improvement (Figures 4). The quadratic model demonstrated a steep initial slope of LVEF improvement at lower doses, with diminishing returns as dose increased. The minimum effective dose producing clinically meaningful LVEF improvement (defined as the lower bound of the 95% prediction interval exceeding zero) was determined to be 280 MET-minutes/week. The optimal dose range, corresponding to the plateau phase of the dose-response curve, was identified between 500 and 800 MET-minutes/week, with the maximum predicted LVEF improvement of 9.8% (95% CrI: 6.2% to 13.4%) achieved at 670 MET-minutes/week.

#### 3.6.3 Dose-response relationships by exercise modality

**Figure 5:**
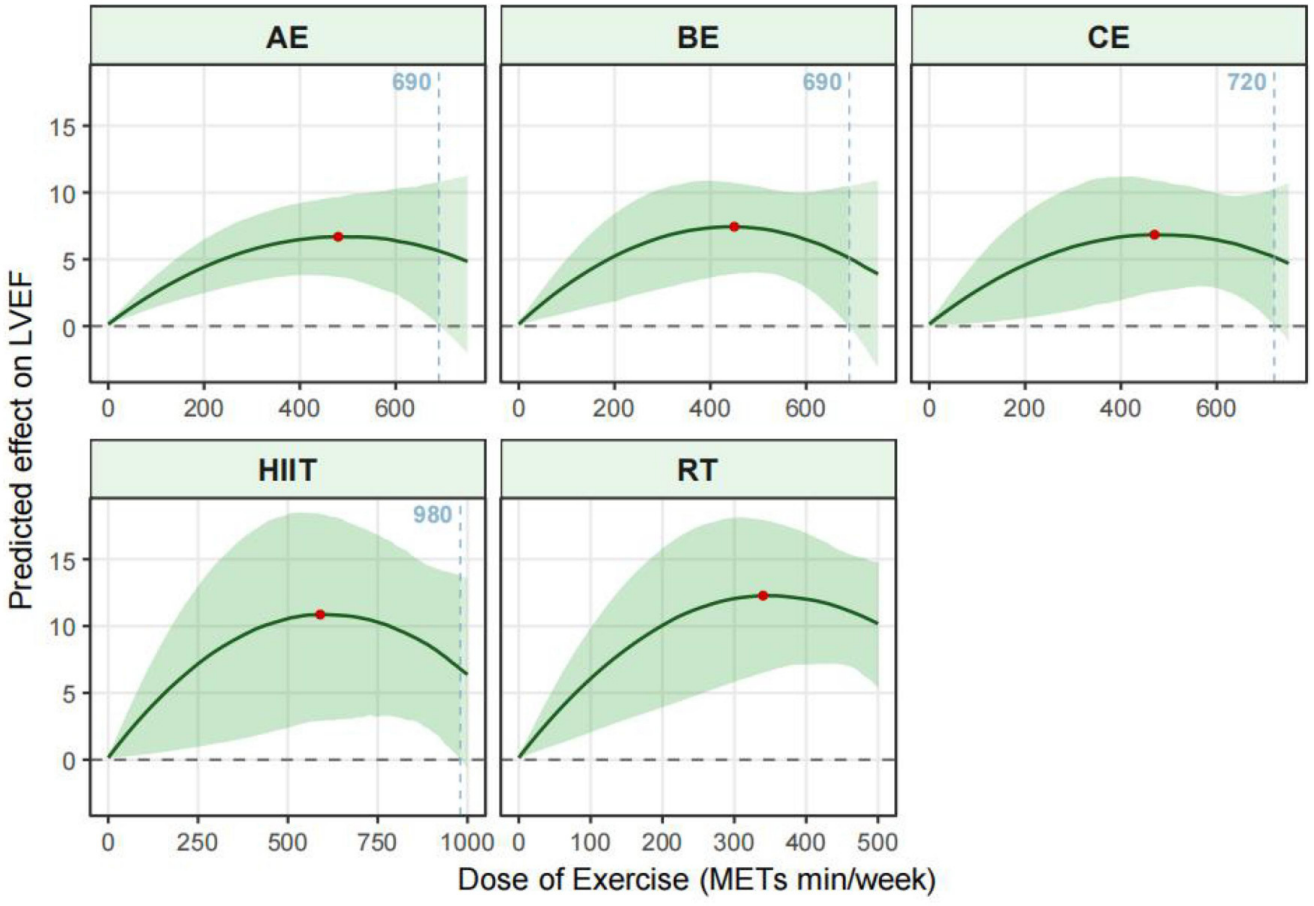
Dose-response relationship between different exercise modalities and LVEF levels in patients with heart failure.the blue area represents the significant 95% credible interval, and the blue numbers indicate the specific dose values (MET-minutes/week) at the start and end points.

Distinct dose-response patterns were observed across exercise modalities (Figures 5). Resistance training exhibited the steepest initial slope, with significant LVEF improvement observed at doses as low as 220 MET-minutes/week. The optimal dose range for resistance training was 330–440 MET-minutes/week, yielding predicted improvements of 8.2%–9.1% (Supplementary Table S9). Detailed summaries are provided in Supplementary Appendix 10.

In contrast to resistance training, the dose-response curve for HIIT was shifted toward higher doses, requiring higher doses to achieve comparable effects. Significant improvement emerged at 440 MET-minutes/week, with optimal effects observed at 560–780 MET-minutes/week (improvement: 8.5%–8.9%).

Combined aerobic and resistance training demonstrated a more gradual dose-response relationship, with significant benefits appearing at 330 MET-minutes/week and maximal effects achieved at 500–670 MET-minutes/week (improvement: 6.5%–7.2%). Aerobic exercise alone displayed the flattest dose-response curve, with significant improvement from 250 MET-minutes/week and optimal effects at 420–580 MET-minutes/week (improvement: 5.1%–5.8%). Mind-body exercise showed a relatively modest dose-response relationship, requiring at least 330 MET-minutes/week for clinically meaningful improvement, with maximal effects at 420–500 MET-minutes/week (improvement: 4.9%–5.3%).

#### 3.6.4 Comparative efficacy across dose levels

Treatment rankings incorporating dose levels (Supplementary Table S12, Figure S13) revealed that resistance training at 330–440 MET-minutes/week consistently ranked as the most effective intervention (mean rank: 4.8–6.3), followed by HIIT at 560–780 MET-minutes/week (mean rank: 9.2–11.1). Lower-dose resistance training (220–280 MET-minutes/week) maintained favorable rankings (mean rank: 6.7–10.1), whereas high-dose aerobic exercise (670–750 MET-minutes/week) and mind-body exercise (580–750 MET-minutes/week) demonstrated substantially lower rankings (mean rank: 27.4–36.2). Detailed summaries are provided in Supplementary Appendix 10.

### 3.7 Sensitivity analyses and meta-regression

#### 3.7.1 Sensitivity analysis

Exclusion of studies at high risk of bias did not substantially alter study findings (Supplementary Table S10). The treatment hierarchy remained stable, with resistance training retaining the highest SUCRA (92.5%), followed by HIIT (75.8%), and comparative effects between exercise modalities following consistent patterns (Supplementary Figure S13a, Table S11). The complete league table for this sensitivity analysis is provided in Supplementary Table S6. Similarly, exclusion of studies with intervention duration <4 weeks (removing 5 studies, 8 data points) yielded consistent results (Supplementary Table S12, Figure S13b). Resistance training maintained its superior ranking (SUCRA: 94.1%) and significant advantage over aerobic exercise (MD: 4.81%, 95% CrI: 1.82 to 7.77). Dose-response patterns remained unchanged in both sensitivity analyses; summaries are provided in Supplementary Appendix 11.

#### 3.7.2 Network meta-regression

Univariate network meta-regression was performed to examine potential moderating effects of participant and intervention characteristics (Supplementary Table S12). None of the covariates examined significantly modified treatment effects, including age (*β*: 2.22, 95% CrI: −0.68–5.02), intervention duration (*β*: −1.34, 95% CrI: −4.60–1.81), exercise frequency (*β*: 1.09, 95% CrI: −1.75–3.78), publication year (*β*: 0.33, 95% CrI: −2.86–3.46), proportion of male participants (*β*: 0.28, 95% CrI: −2.88–3.63), or sample size (*β*: 0.34, 95% CrI: −3.00–3.49). Model fit was not improved by covariate inclusion compared with the unadjusted model, and between-study heterogeneity remained similar across all meta-regression analyses.

## 4 Discussion

### 4.1 Principal findings in the context of heart failure rehabilitation

This study, through Bayesian network meta-analysis (NMA) and model-based dose-response network meta-analysis (MBNMA), systematically quantified the relative efficacy of different exercise modalities on left ventricular ejection fraction (LVEF) in patients with heart failure and their dose-response relationships. The principal findings are summarized in the Results section; here we focus on interpretation and mechanistic understanding within the specific context of contemporary heart failure management.

It is essential to acknowledge at the outset that the included trials encompassed a heterogeneous heart failure population, spanning heart failure with reduced ejection fraction (HFrEF), heart failure with preserved ejection fraction (HFpEF), and mixed phenotypes with varying etiologies (ischemic, non-ischemic, hypertensive) and severity levels (NYHA class I–IV). This heterogeneity, while enhancing external validity, introduces complexity in interpreting modality-specific effects, as exercise responses differ markedly between HFrEF and HFpEF phenotypes (22). HFrEF is characterized by impaired systolic function, neurohormonal activation, and peripheral muscle deconditioning, whereas HFpEF primarily involves diastolic dysfunction, ventricular stiffness, and impaired chronotropic reserve (23). These divergent pathophysiological substrates likely modulate the efficacy of distinct exercise modalities, a consideration that underlies our cautious interpretation of pooled estimates.

Resistance training demonstrated the largest network-estimated effect on LVEF (MD: 10.0; 95% CrI: 6.5, 13.0; SUCRA: 93.1%). However, this estimate was based on only five direct studies, with substantial contribution from indirect evidence. The low number of RT studies limits estimation precision and introduces uncertainty regarding whether RT is genuinely superior across all heart failure phenotypes or whether the ranking reflects sparse-data bias in SUCRA probabilities. Previous methodological investigations have demonstrated that interventions supported by few small studies can achieve high SUCRA values that may not stabilize with additional evidence (24). Therefore, while RT shows promise in heart failure populations, the evidence base remains insufficient for definitive, phenotype-specific conclusions.

The superior ranking of RT may be explained through skeletal muscle-heart cross-talk mechanisms that are particularly relevant in heart failure. Morris et al. (2024) conducted a comprehensive systematic review of resistance training in heart failure, demonstrating that RT improves not only LVEF (weighted increase: 24.8 ± 10.9%) but also peak VO₂, 6-minute walk distance, diastolic function (E/e′), and quality of life, with only one serious adverse event reported across all included trials. The mechanisms underlying these benefits include reversal of the adverse muscle phenotype characteristic of heart failure (shift from fast-twitch glycolytic to slow-twitch oxidative fibers), reduction in systemic inflammation (TNF-α, IL-6), and enhancement of endothelial function (25). Xi et al. further demonstrated that dynamic resistance exercise promotes myocardial angiogenesis through activation of the skeletal muscle-derived FSTL1-DIP2A-Smad2/3 signaling pathway, a mechanism that may be particularly salient in ischemic cardiomyopathy where microvascular rarefaction contributes to disease progression. However, the relative contribution of these mechanisms in HFrEF versus HFpEF remains uncertain, and this explanation should be regarded as speculative pending phenotype-specific validation.

HIIT ranked second (MD: 8.4; 95% CrI: 4.2, 12.0; SUCRA: 76.1%) but was supported by only three studies (156 participants), rendering this ranking highly uncertain. In heart failure populations, HIIT has demonstrated particular efficacy for improving cardiorespiratory fitness. A 2024 systematic review and meta-analysis by Hua et al. identified center-based cardiac rehabilitation with HIIT as the most effective modality for enhancing LVEF in chronic heart failure, while combined aerobic-resistance training optimized quality of life and reduced rehospitalization (26). Similarly, a 2024 narrative review of 18 studies (3,401 patients) found that HIIT tended to produce the largest short-term VO₂peak gains in HFrEF, with sustained benefits on ventilatory efficiency and quality of life (27). The higher dose threshold required for HIIT benefits in our analysis (440 MET-minutes/week) may reflect the need for substantial sympathetic and metabolic stimulation to overcome the peripheral muscle pathology and endothelial dysfunction characteristic of chronic heart failure (28). However, the safety of HIIT in advanced NYHA class III–IV patients remains debated, and our findings should not be extrapolated to this high-risk subgroup without hemodynamic monitoring.

Notably, combined aerobic and resistance training (CE) ranked lower (SUCRA: 46.0%) than RT alone in this analysis. This finding appears to conflict with the 2024 network meta-analysis by Hua et al., which identified combined cardiac rehabilitation (aerobic + resistance) as the most effective intervention for improving quality of life (MLHFQ) and 6-minute walk test performance, with significant reduction in rehospitalization rates (26). This discrepancy likely arises from heterogeneity in combined training protocol designs across included studies: some employed simple superposition of low-intensity aerobic and low-intensity resistance training without achieving synergistic effects, whereas others optimized component sequencing and dose allocation. The 2024 narrative review by Gevaert et al. similarly noted that combined endurance and strength training, when properly structured, offers the most sustainable benefits for HFrEF patients (27). Our findings suggest that the total dose allocation in combined training may result in insufficient dosing of individual components to reach optimal effect ranges, particularly for resistance training (330–440 MET-minutes/week). Future heart failure trials should optimize component ratio and timing arrangement to achieve true synergistic enhancement rather than simple dose dilution.

### 4.2 Dose-Response relationships and clinical implications for heart failure management

The dose-response analysis revealed a non-linear quadratic relationship between exercise dose and LVEF improvement in heart failure patients, with important clinical translational value. The overall minimum effective dose was 280 MET-minutes/week, with an optimal range of 500–800 MET-minutes/week. These thresholds align broadly with the World Health Organization (WHO) 2020 physical activity guidelines, which recommend 150–300 min/week of moderate-intensity aerobic activity or equivalent combinations for adults including those with chronic conditions. However, this study further refines the specific dose requirements for different exercise types in heart failure, providing quantitative evidence for precision implementation of guideline recommendations.

The dose-response curve for RT exhibits an “early plateau” characteristic: significant LVEF improvement at doses as low as 220 MET-minutes/week, with an optimal range of 330–440 MET-minutes/week. This carries dual clinical significance for heart failure management. First, it provides feasibility for deconditioned patients: heart failure patients, particularly those with HFrEF and advanced NYHA class, often have difficulty tolerating large-dose exercise due to severe fatigue and dyspnea; the low-dose efficacy of RT makes it an ideal initial intervention (29). Morris et al. (2024) demonstrated that starting RT slowly and progressing to 80% of one-repetition maximum offers optimal benefit with excellent safety profile (30). Second, it suggests that RT may exhibit a “ceiling effect” in heart failure patients—further increasing load beyond the optimal dose may not yield additional LVEF benefits and may instead increase cardiovascular risk due to excessive pressure loading, particularly in HFpEF where afterload sensitivity is pronounced (31). This contrasts with the dose-response curves for aerobic exercise and HIIT, which maintain relatively gentle upward slopes at higher dose ranges.

However, clinical recommendations must be tempered by evidence certainty limitations. CINeMA ratings indicated very low to moderate certainty across comparisons, primarily limited by within-study bias and imprecision. The marked study imbalance (AE: 22 studies; RT: 5; HIIT: 3) means that modalities with fewer contributing studies rely disproportionately on indirect comparisons, introducing uncertainty into rankings. Furthermore, the heterogeneous heart failure population (HFrEF, HFpEF, mixed etiologies) likely introduces effect modification that our meta-regression could not fully capture due to limited reporting of phenotype-specific data in primary studies. Therefore, rather than positioning RT as a universal “core” intervention for all heart failure patients, we suggest it represents a promising modality warranting cautious clinical implementation with phenotype stratification and prioritization in future heart failure rehabilitation research.

For high-risk heart failure patients with poor baseline cardiac function and extensive comorbidity burden, low-dose RT (220–330 MET-minutes/week) may be considered given its favorable initial slope, with careful hemodynamic monitoring. For younger heart failure patients with better cardiac reserve, HIIT (560–780 MET-minutes/week) may be pursued to achieve substantial functional improvements, acknowledging the limited evidence base. Aerobic exercise (420–580 MET-minutes/week) and mind-body exercise (420–500 MET-minutes/week) remain viable alternatives with more established safety profiles, particularly for HFpEF patients where diastolic function improvement may be more clinically relevant than systolic augmentation.

The dose parameters identified can be directly translated into clinically applicable exercise prescriptions for heart failure cardiac rehabilitation. For RT, 330–440 MET-minutes/week corresponds to: 2–3 sessions per week, 30–40 min per session, at moderate intensity (4.5 MET); or 2–3 sessions per week, 20–25 min per session, at vigorous intensity (6.0 MET) circuit training. These parameters align with Fisher et al.'s systematic review of RT in heart failure patients, which confirmed that RT safely improves peak oxygen uptake (VO₂peak) and 6-minute walk distance (6MWD) without adverse effects on left ventricular parameters (32). However, phenotype-specific validation (HFrEF vs. HFpEF) remains necessary.

### 4.3 Phenotype-specific considerations: HFrEF versus HFpEF

A critical interpretive consideration is the differential response to exercise training between heart failure with reduced ejection fraction (HFrEF) and heart failure with preserved ejection fraction (HFpEF). Although the present analysis pooled these phenotypes due to limited phenotype-specific reporting in primary studies, emerging evidence suggests markedly divergent exercise responses that have important implications for precision rehabilitation.

In HFrEF, exercise training primarily targets: (1) reversal of peripheral muscle deconditioning and shift from glycolytic to oxidative fiber type; (2) modulation of neurohormonal overactivation; (3) enhancement of endothelial function and nitric oxide bioavailability; and (4) improvement of cardiac systolic function through favorable remodeling (33).

A large-scale prospective study based on the UK Biobank, using wrist-worn accelerometers to objectively quantify physical activity in patients with atrial fibrillation, found that moderate-intensity physical activity was significantly associated with reduced risks of all-cause mortality, incident heart failure, and stroke. Notably, although the study did not directly distinguish between HFpEF and HFrEF, atrial fibrillation—as one of the most common comorbidities of HFpEF—shares substantial population overlap with HFpEF. The risk-benefit relationship curve observed in the study suggests that very high-intensity activity confers no additional protection and even shows a trend toward increased risk in certain subgroups, which is highly consistent with the pathophysiological feature of afterload sensitivity in HFpEF. Furthermore, clinical practice has observed that when patients with HFpEF self-report exercise adherence, they often unintentionally exceed safe limits by overlooking early warning signs such as exertional dyspnea; reliance on subjective reporting rather than objective monitoring may severely underestimate their actual risk (34).

These phenotypic differences have direct implications for exercise prescription. In HFrEF, where systolic augmentation is the primary therapeutic target, RT and HIIT may offer particular advantage through their distinct mechanisms: RT via myokine-mediated angiogenesis and muscle fiber type transformation, and HIIT via sympathetic modulation and peripheral oxidative capacity enhancement. In HFpEF, where diastolic function and afterload sensitivity predominate, moderate-intensity aerobic exercise and mind-body exercises may be preferable, as they improve ventricular compliance and autonomic balance without excessive hemodynamic stress. The 2024 study by Gevaert et al. further highlighted that HIIT and moderate continuous training did not significantly differ in their effects on nitric oxide metabolites in either HFrEF or HFpEF after 3 and 12 months, suggesting that the choice between these modalities may depend on patient preference and tolerability rather than distinct mechanistic pathways.

Our findings should be interpreted with caution regarding phenotype-specific applicability. The predominance of HFrEF trials in the evidence base (82 of 93 studies in the 2022 meta-analysis) means that RT's superior ranking may primarily reflect efficacy in systolic dysfunction rather than diastolic impairment. Similarly, the limited number of HFpEF-specific trials constrains confident extrapolation of dose-response relationships to this growing patient population. Future network meta-analyses should stratify by heart failure phenotype when primary studies provide adequate reporting.

### 4.4 Limitations

Several limitations warrant consideration when interpreting the present findings in the context of heart failure management.

First, study imbalance and evidence quality. Although 31 RCTs provided a substantial evidence base, the distribution was highly skewed (AE: 22 studies; RT: 5; HIIT: 3). Modalities with fewer studies rely disproportionately on indirect comparisons, and their SUCRA rankings may be unstable. CINeMA ratings ranged from very low to moderate certainty across comparisons, primarily limited by within-study bias (most studies rated “some concerns”) and imprecision. The low number of RT studies constrains estimation precision; additional high-quality RCTs are needed to consolidate these findings.

Second, heart failure phenotype heterogeneity. The included trials encompassed HFrEF, HFpEF, and mixed phenotypes with varying etiologies and severity levels. Exercise responses differ substantially between these subgroups, yet our meta-regression could not adequately account for phenotype-specific effects due to incomplete reporting in primary studies. The predominance of HFrEF trials in the evidence base means that our findings may not be generalizable to HFpEF, where diastolic function rather than systolic augmentation is the primary therapeutic target.

Third, potential inaccuracies in dose calculations. Despite our detailed MET assignment protocol, incomplete reporting of exercise parameters in original publications may have introduced dose estimation errors. Resistance training MET values are particularly uncertain because most studies did not report %1RM or direct metabolic measurement. Our assignments relied on RPE scales, repetition ranges, and intensity categories, which involve subjective judgment and may misclassify actual metabolic costs—especially for HIIT and RT. Although we attempted to contact corresponding authors, the 35% response rate necessitated inference for most studies.

Fourth, language and publication bias. Restricting searches to English and Chinese may have excluded relevant trials published in other languages, potentially introducing language bias. The predominance of English-language research may also reflect publication bias favoring positive results.

Fifth, limited direct comparisons. Certain pairwise comparisons (e.g., RT vs. HIIT) had no direct studies, relying entirely on indirect evidence through common comparators, which increases uncertainty.

Sixth, outcome limitations. LVEF was the sole outcome measure, providing an incomplete picture of heart failure pathophysiology. It does not capture diastolic function (particularly relevant in HFpEF), quality of life, exercise capacity, ventilatory efficiency, neurohormonal biomarkers (BNP/NT-proBNP), or long-term prognostic outcomes—dimensions that may be more clinically meaningful than subacute LVEF changes. The lack of data on heart failure hospitalization, cardiovascular mortality, or all-cause mortality limits the clinical utility of findings for prognostic decision-making.

## 5 Conclusion

This study indicates that resistance training may be the most effective exercise modality for improving left ventricular ejection fraction in heart failure patients, with favorable effects at low to moderate doses (330–440 MET-minutes/week). However, due to limited evidence and imbalance in studies across exercise modalities, the conclusion should be interpreted with caution. The heterogeneous responses across exercise modalities support the concept of precision rehabilitation. Future research requires more high-quality randomized controlled trials, as well as standardized exercise reporting and assessment of broader outcome measures.

## Data Availability

The original contributions presented in the study are included in the article/Supplementary Material, further inquiries can be directed to the corresponding authors.
