# Supplementary material for: Exercise modalities and dose-response for LVEF Improvement in heart failure patients: a systematic review and network meta-analysis
Source: Front Cardiovasc Med. 2026 Jun 5;13:1834798. doi: 10.3389/fcvm.2026.1834798 (PMC13278864; doi:10.3389/fcvm.2026.1834798)
Supplement: Supplementary file 1 [file Datasheet1.docx]

***Supplementary Material***

[Appendix 1. Search strategies 2](#_Toc21167)

[Appendix 2. Characteristics of included studies 7](#_Toc20714)

[Appendix 3. Summary of risk of bias 13](#_Toc17861)

[Appendix 4. Evidence assessment 14](#_Toc12601)

[Appendix 5. Pairwise meta-analyses 15](#_Toc1727)

[Appendix 6. Bayesian network meta-analysis 17](#_Toc7353)

[Appendix 7. Forest plot of all studies 20](#_Toc15515)

[Appendix 8. Model Selection and Fitting 22](#_Toc1844)

[Appendix 9. Predictions ranking 32](#_Toc31131)

[Appendix 10. Sensitivity analysis 35](#_Toc30978)

# Appendix 1. Search strategies

| Databases | Terms |
| --- | --- |
| PubMed | #1 "Heart Failure"[MeSH]  #2 "Cardiac Failure"[Title/Abstract] OR "Congestive Heart Failure"[Title/Abstract] OR "Heart Decompensation"[Title/Abstract] OR "Heart Failure, Congestive"[Title/Abstract] OR "Heart Failure, Left-Sided"[Title/Abstract] OR "Heart Failure, Right-Sided"[Title/Abstract] OR "Left-Sided Heart Failure"[Title/Abstract] OR "Myocardial Failure"[Title/Abstract] OR "Right-Sided Heart Failure"[Title/Abstract]  #3 #1 OR #2  #4 "Exercise"[MeSH] OR "Physical Exertion"[MeSH] OR "Exercise Therapy"[MeSH] OR "Resistance Training"[MeSH] OR "High-Intensity Interval Training"[MeSH] OR "exercise"[Title/Abstract] OR "physical activity"[Title/Abstract] OR "aerobic training"[Title/Abstract] OR "aerobic exercise"[Title/Abstract] OR "endurance training"[Title/Abstract] OR "endurance exercise"[Title/Abstract] OR "resistance training"[Title/Abstract] OR "resistance exercise"[Title/Abstract] OR "strength training"[Title/Abstract] OR "strength exercise"[Title/Abstract] OR "combined training"[Title/Abstract] OR "combined exercise"[Title/Abstract] OR "concurrent training"[Title/Abstract] OR "interval training"[Title/Abstract] OR "interval exercise"[Title/Abstract] OR "sprint training"[Title/Abstract]  #5 #3 AND #4  #6 "Ventricular Function, Left"[MeSH] OR "Stroke Volume"[MeSH] OR "Echocardiography"[MeSH] OR "ventricular function, left"[Title/Abstract] OR "left ventricular function"[Title/Abstract] OR "left ventricle function"[Title/Abstract] OR "LV function"[Title/Abstract] OR "cardiac function"[Title/Abstract] OR "heart function"[Title/Abstract] OR "systolic function"[Title/Abstract] OR "left ventricular systolic function"[Title/Abstract] OR "LV systolic function"[Title/Abstract] OR "ejection fraction"[Title/Abstract] OR "ejection fractions"[Title/Abstract] OR "fraction, ejection"[Title/Abstract] OR "fractions, ejection"[Title/Abstract] OR "ventricular ejection fraction"[Title/Abstract] OR "ejection fraction, ventricular"[Title/Abstract] OR "left ventricular ejection fraction"[Title/Abstract] OR "ejection fraction, left ventricular"[Title/Abstract] OR "LVEF"[Title/Abstract] OR "LV ejection fraction"[Title/Abstract] OR "left ventricle ejection fraction"[Title/Abstract] OR "ejection fraction, left ventricle"[Title/Abstract]  #7 #5 AND #6  #8 ("randomized controlled trial"[Publication Type] OR "randomized controlled trials as topic"[MeSH] OR "randomized"[Title/Abstract] OR "randomised"[Title/Abstract] OR "randomly"[Title/Abstract] OR "trial"[Title/Abstract] OR "placebo"[Title/Abstract]) |
| Embase | ('Heart Failure'/exp OR 'Cardiac Failure':ti,ab OR 'Congestive Heart Failure':ti,ab OR 'Heart Decompensation':ti,ab OR 'Heart Failure, Congestive':ti,ab OR ' Heart Failure, Left-Sided':ti,ab OR 'Heart Failure, Right-Sided':ti,ab OR 'Left-Sided Heart Failure':ti,ab OR 'Myocardial Failure':ti,ab OR 'Right-Sided Heart Failure':ti,ab ) AND ('exercise'/exp OR 'physical exertion'/exp OR 'exercise therapy'/exp OR 'resistance training'/exp OR 'high intensity interval training'/exp OR 'exercise':ti,ab OR 'physical activity':ti,ab OR 'aerobic training':ti,ab OR 'aerobic exercise':ti,ab OR 'endurance training':ti,ab OR 'endurance exercise':ti,ab OR 'resistance training':ti,ab OR 'resistance exercise':ti,ab OR 'strength training':ti,ab OR 'strength exercise':ti,ab OR 'combined training':ti,ab OR 'combined exercise':ti,ab OR 'concurrent training':ti,ab OR 'interval training':ti,ab OR 'interval exercise':ti,ab OR 'sprint training':ti,ab) AND ('left ventricular function'/exp OR 'stroke volume'/exp OR 'echocardiography'/exp OR 'ventricular function, left':ti,ab OR 'left ventricular function':ti,ab OR 'left ventricle function':ti,ab OR 'LV function':ti,ab OR 'cardiac function':ti,ab OR 'heart function':ti,ab OR 'systolic function':ti,ab OR 'left ventricular systolic function':ti,ab OR 'LV systolic function':ti,ab OR 'ejection fraction':ti,ab OR 'ejection fractions':ti,ab OR 'fraction, ejection':ti,ab OR 'fractions, ejection':ti,ab OR 'ventricular ejection fraction':ti,ab OR 'ejection fraction, ventricular':ti,ab OR 'left ventricular ejection fraction':ti,ab OR 'ejection fraction, left ventricular':ti,ab OR 'LVEF':ti,ab OR 'LV ejection fraction':ti,ab OR 'left ventricle ejection fraction':ti,ab OR 'ejection fraction, left ventricle':ti,ab) AND (('randomized controlled trial'/exp OR 'controlled clinical trial'/exp OR 'randomization'/exp OR 'double blind procedure'/exp OR 'single blind procedure'/exp OR 'placebo'/exp OR 'crossover procedure'/exp OR 'multicenter study'/exp) OR (random*:ti,ab OR placebo:ti,ab OR ((single*:ti,ab OR double*:ti,ab OR triple*:ti,ab OR trebl*:ti,ab) NEAR/3 (blind*:ti,ab OR mask*:ti,ab)))) |
| The Cochrane Library | #1 MeSH descriptor: [Heart Failure] explode all trees  #2 ("Cardiac Failure" OR "Congestive Heart Failure" OR "Heart Decompensation" OR "Heart Failure, Congestive" OR "Heart Failure, Left-Sided" OR "Heart Failure, Right-Sided" OR "Left-Sided Heart Failure" OR "Myocardial Failure" OR "Right-Sided Heart Failure"):ti,ab,kw  #3 #1 OR #2  #4 MeSH descriptor: [Exercise] explode all trees  #5 MeSH descriptor: [Physical Exertion] explode all trees  #6 MeSH descriptor: [Exercise Therapy] explode all trees  #7 MeSH descriptor: [Resistance Training] explode all trees  #8 MeSH descriptor: [High-Intensity Interval Training] explode all trees  #9 ("exercise" OR "physical activity" OR "aerobic training" OR "aerobic exercise" OR "endurance training" OR "endurance exercise" OR "resistance training" OR "resistance exercise" OR "strength training" OR "strength exercise" OR "combined training" OR "combined exercise" OR "concurrent training" OR "interval training" OR "interval exercise" OR "sprint training"):ti,ab,kw  #10 #4 OR #5 OR #6 OR #7 OR #8 OR #9  #11 #3 AND #10  #12 MeSH descriptor: [Ventricular Function, Left] explode all trees  #13 MeSH descriptor: [Stroke Volume] explode all trees  #14 MeSH descriptor: [Echocardiography] explode all trees  #15 ("ventricular function, left" OR "left ventricular function" OR "left ventricle function" OR "LV function" OR "cardiac function" OR "heart function" OR "systolic function" OR "left ventricular systolic function" OR "LV systolic function" OR "ejection fraction" OR "ejection fractions" OR "fraction, ejection" OR "fractions, ejection" OR "ventricular ejection fraction" OR "ejection fraction, ventricular" OR "left ventricular ejection fraction" OR "ejection fraction, left ventricular" OR "LVEF" OR "LV ejection fraction" OR "left ventricle ejection fraction" OR "ejection fraction, left ventricle"):ti,ab,kw  #16 #12 OR #13 OR #14 OR #15  #17 #11 AND #16 |
| Web of Science | TS=(("Heart Failure" OR "Cardiac Failure" OR "Congestive Heart Failure" OR "Heart Decompensation" OR "Heart Failure, Congestive"OR " Heart Failure, Left-Sided"OR "Heart Failure, Right-Sided"OR "Left-Sided Heart Failure"OR "Myocardial Failure"OR "Right-Sided Heart Failure") AND ("exercise" OR "physical activity" OR "aerobic training" OR "aerobic exercise" OR "endurance training" OR "endurance exercise" OR "resistance training" OR "resistance exercise" OR "strength training" OR "strength exercise" OR "combined training" OR "combined exercise" OR "concurrent training" OR "interval training" OR "interval exercise" OR "sprint training") AND ("left ventricular function" OR "left ventricle function" OR "LV function" OR "cardiac function" OR "heart function" OR "systolic function" OR "left ventricular systolic function" OR "LV systolic function" OR "ejection fraction" OR "ejection fractions" OR "ventricular ejection fraction" OR "left ventricular ejection fraction" OR "LVEF" OR "LV ejection fraction" OR "left ventricle ejection fraction")) AND TS=(random* OR "clinical trial*" OR "controlled trial*" OR placebo OR "single blind*" OR "double blind*" OR "randomized controlled trial" OR "randomised controlled trial") |
| China National Knowledge Infrastructure | (SU='心脏衰竭' OR SU='充血性心力衰竭' OR SU='心脏失代偿' OR SU='心力衰竭，充血' OR SU='心力衰竭，左侧' OR SU='右侧心力衰竭' OR SU='左侧心力衰竭' OR SU='心肌衰竭' OR SU='右侧心力衰竭' ) AND (SU='运动' OR SU='体育锻炼' OR SU='有氧运动' OR SU='有氧训练' OR SU='耐力运动' OR SU='耐力训练' OR SU='抗阻运动' OR SU='抗阻训练' OR SU='力量训练' OR SU='力量练习' OR SU='间歇运动' OR SU='间歇训练' OR SU='高强度间歇训练' OR TI='运动' OR TI='体育锻炼' OR TI='有氧运动' OR TI='有氧训练' OR TI='耐力运动' OR TI='耐力训练' OR TI='抗阻运动' OR TI='抗阻训练' OR TI='力量训练' OR TI='力量练习' OR TI='间歇运动' OR TI='间歇训练' OR TI='高强度间歇训练' OR AB='运动' OR AB='体育锻炼' OR AB='有氧运动' OR AB='有氧训练' OR AB='耐力运动' OR AB='耐力训练' OR AB='抗阻运动' OR AB='抗阻训练' OR AB='力量训练' OR AB='力量练习' OR AB='间歇运动' OR AB='间歇训练' OR AB='高强度间歇训练') AND (SU='左心室功能' OR SU='心功能' OR SU='心脏功能' OR SU='收缩功能' OR SU='左心室收缩功能' OR SU='射血分数' OR SU='左室射血分数' OR SU='LVEF' OR TI='左心室功能' OR TI='心功能' OR TI='心脏功能' OR TI='收缩功能' OR TI='左心室收缩功能' OR TI='射血分数' OR TI='左室射血分数' OR TI='LVEF' OR AB='左心室功能' OR AB='心功能' OR AB='心脏功能' OR AB='收缩功能' OR AB='左心室收缩功能' OR AB='射血分数' OR AB='左室射血分数' OR AB='LVEF') AND (SU='随机' OR TI='随机' OR AB='随机' OR FT='随机') |
| Wanfang Database | ("心脏衰竭"+"充血性心力衰竭"+"心脏失代偿"+"心力衰竭，充血"+"心力衰竭，左侧"+"右侧心力衰竭"+"左侧心力衰竭"+"心肌衰竭"+"右侧心力衰竭") * 主题:("运动"+"体育锻炼"+"有氧运动"+"有氧训练"+"耐力运动"+"耐力训练"+"抗阻运动"+"抗阻训练"+"力量训练"+"力量练习"+"间歇运动"+"间歇训练"+"高强度间歇训练") * 主题:("左心室功能"+"心功能"+"心脏功能"+"收缩功能"+"左心室收缩功能"+"射血分数"+"左室射血分数"+"LVEF") AND (SU='随机' OR FT='随机') |
| Weipu  Database | (U=心肌梗死 OR U=心肌梗塞 OR U=心血管卒中 OR U=心脏病发作 OR U=急性心肌梗死 OR U=心梗) AND (U=运动 OR U=体育锻炼 OR U=有氧运动 OR U=有氧训练 OR U=耐力运动 OR U=耐力训练 OR U=抗阻运动 OR U=抗阻训练 OR U=力量训练 OR U=力量练习 OR U=间歇运动 OR U=间歇训练 OR U=高强度间歇训练) AND (U=左心室功能 OR U=心功能 OR U=心脏功能 OR U=收缩功能 OR U=左心室收缩功能 OR U=射血分数 OR U=左室射血分数 OR U=LVEF) |
| Chinese Biomedical Database | (( "心脏衰竭"[常用字段:智能] OR "充血性心力衰竭"[常用字段:智能] OR "心脏失代偿"[常用字段:智能] OR "心力衰竭，充血"[常用字段:智能] OR "心力衰竭，左侧"[常用字段:智能] OR "右侧心力衰竭"[常用字段:智能] OR "左侧心力衰竭"[常用字段:智能] OR "心肌衰竭"[常用字段:智能] OR "右侧心力衰竭"[常用字段:智能])) AND (( "运动"[常用字段:智能] OR "体育锻炼"[常用字段:智能] OR "有氧运动"[常用字段:智能] OR "有氧训练"[常用字段:智能] OR "耐力运动"[常用字段:智能] OR "耐力训练"[常用字段:智能] OR "抗阻运动"[常用字段:智能] OR "抗阻训练"[常用字段:智能] OR "力量训练"[常用字段:智能] OR "力量练习"[常用字段:智能] OR "间歇运动"[常用字段:智能] OR "间歇训练"[常用字段:智能] OR "高强度间歇训练"[常用字段:智能] )) AND (( "心室功能，左"[常用字段:智能] OR "每搏输出量"[常用字段:智能] OR "超声心动描记术"[常用字段:智能] OR "左心室功能"[常用字段:智能] OR "左室功能"[常用字段:智能] OR "心脏功能"[常用字段:智能] OR "心功能"[常用字段:智能] OR "收缩功能"[常用字段:智能] OR "左心室收缩功能"[常用字段:智能] OR "射血分数"[常用字段:智能] OR "左心室射血分数"[常用字段:智能] OR "LVEF"[常用字段:智能] OR "左室射血分数"[常用字段:智能] )) |

# Appendix 2. Characteristics of included studies

**Supplementary Table 2.** Characteristics of included studies

| **First Author (Year)** | **Sample Size (Exp/Con)** | **Age (years)** | **Intervention Type** | **Brief Description of Intervention** |
| --- | --- | --- | --- | --- |
| Austin (2005) | 100 / 100 | Con: 71.8 ± 6.8 | RT | 8-week outpatient cardiac rehabilitation plus 16-week community-based exercise, including aerobic endurance training and low resistance/high repetition muscular strength work, twice weekly. |
|  |  | Exp: 71.9 ± 6.3 | CT |  |
| Belardinelli (1999) | 50 / 49 | Con: 53 ± 9 | AE | 14-month supervised exercise training: initial 8 weeks 3 times/week, then 12 months 2 times/week; each session ~1 hour including stretching and 40 min cycling at 60% peak VO₂. |
|  |  | Exp: 56 ± 7 | CT |  |
| Brubaker (2009) | 30 / 29 | Exp: 70.4 ± 5.3 | AE | 16-week supervised endurance exercise (walking and stationary cycling), 3 times/week, 30-40 min/session, at 60-70% heart rate reserve. |
|  |  | Con: 69.9 ± 6.3 | CT |  |
| Chrysohoou (2015) | 33 / 39 | Exp: 63 ± 9 | HIIT | 12-week high-intensity interval aerobic training (30s at 100% of max workload followed by 30s rest, 45 min/session, 3 days/week) combined with strength training. |
|  |  | Con: 56 ± 11 | CT |  |
| Chen (2014) | 19 / 18 | Exp: 61 ± 11.8 | AE | 3-month home-based cardiac rehabilitation including aerobic exercise (walking, jogging, cycling) at least 3 times/week, 30 min/session, at 60-80% peak HR or Borg scale 12-13. |
|  |  | Con: 60 ± 16 | CT |  |
| Chien (2011) | 24 / 27 | Exp: 57 ± 16 | CE | 8-week individualized home-based exercise program including walking and major muscle group strengthening, at least 30 min/session, 3 times/week, supervised via regular telephone follow-up. |
|  |  | Con: 59 ± 16 | CT |  |
| Chen (2025) | 59/59 | Exp: 69.78 ± 5.59 | RT | Resistance band training (6 exercises, 2-4 sets, 2-3 times/week). |
|  |  | Con: 69.14 ± 5.83 | CT |  |
| Conraads (2004) | 27 / 22 | Exp: 59 ± 2 | CE | 4-month combined endurance (cycling/jogging) and resistance training (9 prescribed exercises at 50-60% of 1-RM), alternating modalities. |
|  |  | Con: 59 ± 2 | CT |  |
| Corvera-Tindel (2004) | 42 / 37 | Exp: 63.8 ± 10.1 | AE | 12-week progressive home walking program, 5 days/week, intensity gradually increased from 40% to 65% of maximal HR. |
|  |  | Con: 61.3 ± 11.1 | CT |  |
| Dalal (2018) | 107 / 109 | Exp: 69.7 ± 10.9 | RT | 12-week home-based cardiac rehabilitation (REACH-HF) including a patient manual (with chair-based and progressive walking programs), an interactive 'Progress Tracker', a 'Family and Friends Resource', and facilitation by cardiac nurses/physiotherapists. |
|  |  | Con: 69.9 ± 11.0 | CT |  |
| Du (2018) | 67 / 65 | Exp: 62 ± 15 | AE | 6-month "Home-Heart-Walk" (HHW) intervention, a self-administered 6-minute walk test performed weekly. |
|  |  | Con: 58 ± 15 | CT |  |
| Dracup (2007) | 86 / 87 | Exp: 53.3 ± 12.7 | CE | 1-year graduated, low-level home exercise program including aerobic walking (gradually increased to 45 min, intensity up to 60% max HR) and resistance training (upper/lower body strengthening). |
|  |  | Con: 54.6 ± 12.5 | CT |  |
| Hambrecht (1995) | 12/10 | Exp: 50 ± 12 | BE | 6-month exercise training: initial 3 weeks supervised in-hospital (6 times/day, 10 min cycle ergometer at 70% peak VO₂), followed by home-based training (2 times/day, 40 min total) plus 2 group sessions/week. |
|  |  | Control: 52 ± 8 | CT |  |
| Hwang (2017) | 24 / 29 | Exp: 68 ± 14 | BE | 12-week real-time, group-based, home-based telerehabilitation via online videoconferencing software, 2 times/week, 60 min/session, including aerobic and strength exercises, intensity gradually increased based on RPE 9-13. |
|  |  | Con: 67 ± 11 | CT |  |
| Giannuzzi (2003) | 45 / 45 | Exp: 60 ± 7 | AE | 6-month supervised continuous cycle ergometer training at 60% of peak VO₂, ≥3 times/week, combined with a home walking program. |
|  |  | Con: 61 ± 7 | CT |  |
| Karapolat (2009) | 37 / 37 | Exp: 45.2 ± 13.6 | AE | 8-week hospital- or home-based exercise program including flexibility, aerobic (treadmill or walking at 60-70% of pVO₂), and breathing exercises. |
|  |  | Con: 44.1 ± 11.5 | CT |  |
| Kitzman (2021) | 175 / 174 | Exp: 73.1 ± 8.5 | CE | Early, transitional, tailored, progressive rehabilitation intervention for older patients hospitalized for acute decompensated heart failure, focusing on four physical function domains (strength, balance, mobility, endurance), initiated during or shortly after hospitalization and continued for 36 outpatient sessions. |
|  |  | Con: 72.2 ± 7.7 | CT |  |
| Kiilavuori (1996) | 12/15 | Exp: 52 ± 7 | AE | 3-month supervised cycle ergometer training at 50-60% of peak VO₂, 30 min/session, 3 times/week, followed by 3 months of home-based training. |
|  |  | Con: 52 ± 9 | CT |  |
| Koukouvou (2004) | 10/16 | Exp: 52.3 ± 9.2 | RT | 6-month supervised exercise training including aerobic exercises (cycling, walking/jogging, calisthenics, etc., at 50-70% peak VO₂, 60 min/session, 3-4 times/week) and resistance exercises added after the first 3 months. |
|  |  | Con: 52.8 ± 10.6 | CT |  |
| Lin (2024) | 55/55 | Exp: 68.59 ± 5.43 | AE | 12-week pulmonary function rehabilitation nursing based on 5E rehabilitation model (Encouragement, Education, Exercise, Employment, Evaluation) |
|  |  | Con: 68.28 ± 5.69 | CT |  |
| Luo (2014) | 20 / 20 / 20 | Exp: 45.7 ± 13.0 | RT | 12-week combined resistance training (50-60% 1RM, 9 exercises, 1-4 sets) plus aerobic cycle ergometer training (50-70% VO₂max, 50 min/session), 3 times/week. |
|  |  | Exp: 50.9 ± 14.6 | AE |  |
|  |  | Con: 49.1 ± 12.5 | CT |  |
| Lundgren (2021) | 31 / 30 | Exp: 67.6 ± 10.9 | HIIT | 3-month real-time, home-based, remotely supervised high-intensity interval training via videoconferencing software, including warm-up, 4x4 min high-intensity intervals (target 85-95% HRmax), and cool-down. |
|  |  | Con: 67.7 ± 11.9 | CT |  |
| Nascimento (2023) | 14/13 | Exp: 63 ± 4.9 | AE | 12-week functional training (FTG) vs. strength training (STG), 3 times/week for 36 sessions. |
|  |  | Con: 58 ± 8.2 | CT |  |
| Nagatomi (2022) | 15 / 15 | Exp: 59.8 ± 10.0 | CE | 3-month comprehensive home-based cardiac rehabilitation using ICT (Fitbit device and app), including patient education, individualized exercise guidance (stretching, resistance, aerobic), and nutritional guidance. |
|  |  | Con: 67.7 ± 8.9 | CT |  |
| Oka (2000) | 20 / 20 | Overall: 30-76 years | CE | 3-month home-based combined walking (3 days/week, intensity gradually increased to ~70% peak HR, 40-60 min/session) and resistance training (2 days/week, total body unilateral exercises, intensity gradually increased to ~75% 1-RM). |
|  |  |  | CT |  |
| O'Connor (2009) | 189 / 198 | Exp: 59.2 (median) | AE | Initially 36 supervised aerobic training sessions (walking, treadmill, or cycling) at 60-70% heart rate reserve, followed by home-based training 5 times/week, 40 min/session. |
|  |  | Con: 59.3 (median) | CT |  |
| Peng (2018) | 49 / 49 | Overall: 66.3 ± 10.5 | HIIT | 8-week home-based telehealth-supervised exercise training (via QQ/WeChat), consisting of 2 phases: Phase 1 endurance (walking/jogging, 3x/week, 20 min/session), Phase 2 adding resistance and strength training (5x/week, 30 min/session), intensity at 40-70% heart rate reserve. |
|  |  |  | CT |  |
| Piotrowicz (2019) | 425 / 425 | Exp: 62.6 ± 10.8 | BE | 9-week hybrid comprehensive telerehabilitation (HCTR): 1 week in-hospital training plus 8 weeks of home-based remote monitoring training (Nordic walking, respiratory muscle training, light resistance exercises). |
|  |  | Con: 62.2 ± 10.2 | CT |  |
| Piotrowicz (2015) | 77 / 34 | Exp: 54.4 ± 10.9 | AE | 8-week home-based telemonitored Nordic walking training, 5 times per week. |
|  |  | Con: 62.1 ± 12.5 | CT |  |
| Piotrowicz (2010) | 77 / 75 | Exp: 56.4 ± 10.9 | AE | 8-week home-based telemonitored walking training, compared to outpatient standard cardiac rehabilitation (interval training on a cycle ergometer). |
|  |  | Con: 60.5 ± 8.8 | CT |  |
| Xueyu (2017) | 40 / 38 | Exp: 78 ± 3.3 | AE | 12-week low-intensity walking protocol including warm-up, walking (target HR: resting HR+5-10 bpm, gradually increased to 30-40 min/day or 1500-3000 steps/day), and cool-down exercises. |
|  |  | Con: 76 ± 4.4 | CT |  |
| Tian (2025) | 44/44 | Exp: 67.59 ± 3.16 | AE | 2-month network-platform-based home cardiac rehabilitation intervention |
|  |  | Con: 67.53 ± 3.12 | CT |  |
| Vetrovsky (2024) | 101 / 101 | Exp: 65.0 (IQR 56.0-72.0) | AE | 6-month lifestyle walking intervention combining self-monitoring with a wrist-worn activity tracker (Garmin vivofit) and monthly telephone counseling from a research nurse. |
|  |  | Exp: 65.0 (IQR 56.0-73.0) | CT |  |
| Xiao (2025) | 71/71 | Exp: 66.58 ± 8.20 | BE | 12-week simplified Tai Chi (Chen-style 18 forms) exercise, 30 min/session, 3-5 times/week. Intensity based on target heart rate (30-60% HRR) and Borg scale. |
|  |  | Con: 65.77 ± 7.20 | CT |  |
| Yao (2025) | 32/32 | Research: 71.78 ± 7.34 | AE | 3-month 6MWT-based rehabilitation training. Exercise intensity prescribed according to 6MWT results, with target heart rate at 40-70% HRR. |
|  |  | Con: 70.23 ± 6.12 | CT |  |
| Yeh (2004) | 15 / 15 | Exp: 66 ± 12 | AE | 12-week Tai Chi training, 1-hour classes twice weekly, including warm-up, 5 simplified Tai Chi movements, and cool-down, with encouragement to practice ≥3 times/week at home. |
|  |  | Con: 61 ± 14 | CT |  |
| Yeh (2011) | 50 / 50 | Exp: 68.1 ± 11.9 | BE | 12-week group-based Tai Chi exercise program, 1-hour classes twice weekly, including warm-up, 5 simplified Tai Chi movements, and cool-down; home practice encouraged. Control group received time-matched health education. |
|  |  | Con: 66.6 ± 12.1 | CT |  |
| Yu (2020) | 100/100 | Exp: 67.7 ± 5.8 | BE | 24-week 24-form simplified Tai Chi, practiced twice daily (morning/afternoon), about 20 min/session. Monitored with wireless physiological parameter devices. |
|  |  | Con: 68.3 ± 6.4 | CT |  |
| Wang (2021) | 31 / 31 | Exp: 60.5 ± 8.0 | BE | 60-day Baduanjin exercise intervention, practiced twice daily, 2 sets/time, each set lasting 8-10 min. Compared with a walking group and a blank control group receiving basic treatment. |
|  |  | Exp: 59.6 ± 10.2 | AE |  |
|  |  | Con: 62.1 ± 9.1 | CT |  |
| Wang (2022) | 33 / 36 | Exp: 63.12 ± 8.89 | BE | 12-week 24-form simplified Tai Chi (high stance, knee angle >150°), 30 min/session, 3-5 times/week. Intensity at 30-60% HRR combined with Borg Scale (11-13). |
|  |  | Con: 64.86 ± 8.17 | CT |  |
| Willenheimer (2001) | 17 / 20 | Exp: 64 ± 5 | AE | 16-week cycle ergometer interval training at 80% of maximal intensity, 3 times/week, followed by a 6-month extended follow-up without intervention (this report is the follow-up). |
|  |  | Con: 64 ± 8 | CT |  |
| Zhang (2004) | 34/36 | Exp: 50.2 ± 13.9 | AE | 3-week exercise training program including cycle ergometer interval training (30s work/60s rest), treadmill walking (3 times/week), and daily ground walking (≥30 min). |
|  |  | Con: 50.4 ± 14.3 | CT |  |

# Appendix 3. Summary of risk of bias


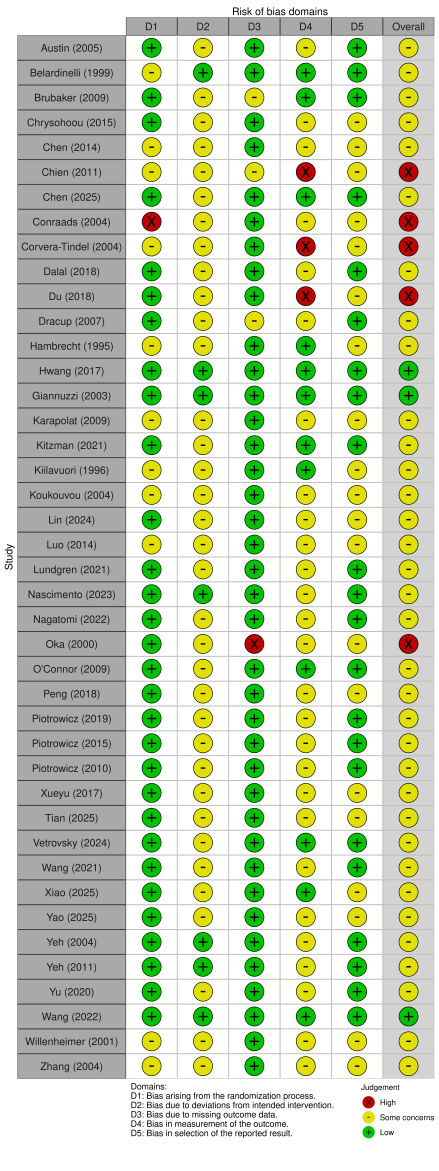


**Supplementary Figure 1.** Summary of risk of bias

# Appendix 4. Evidence assessment


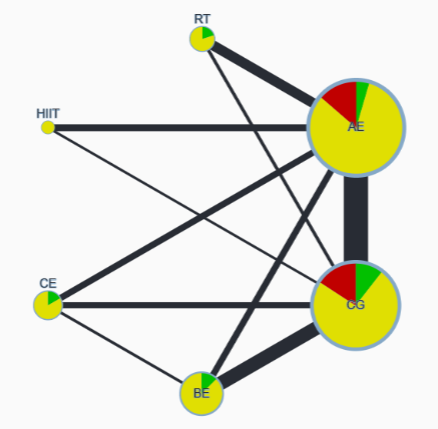


**Supplementary Figure 2.** Evidence assessment

# Appendix 5. Pairwise meta-analyses

**Supplementary Table 1.** League table

| **Outcome** | **Comparison** | | | | |
| --- | --- | --- | --- | --- | --- |
| AE | BE | CE | HIIT | RT | CG |
| AE | 1.47 (-1.33, 4.25) | 0.85 (-2.11, 3.79) | 3.22 (-0.65, 7.01) | 4.79 (1.70, 7.86) | -5.14 (-7.01, -3.30) |
| -1.47 (-4.25, 1.33) | BE | -0.63 (-4.13, 2.88) | 1.74 (-2.91, 6.38) | 3.31 (-0.73, 7.31) | -6.62 (-9.18, -4.06) |
| -0.85 (-3.79, 2.11) | 0.63 (-2.88, 4.13) | CE | 2.36 (-2.38, 7.12) | 3.94 (-0.24, 8.15) | -5.99 (-8.99, -2.97) |
| -3.22 (-7.01, 0.65) | -1.74 (-6.38, 2.91) | -2.36 (-7.12, 2.38) | HIIT | 1.57 (-3.28, 6.42) | -8.37 (-12.45, -4.26) |
| -4.79 (-7.86, -1.70) | -3.31 (-7.31, 0.73) | -3.94 (-8.15, 0.24) | -1.57 (-6.42, 3.28) | RT | -9.93 (-13.34, -6.55) |
| 5.14 (3.30, 7.01) | 6.62 (4.06, 9.18) | 5.99 (2.97, 8.99) | 8.37 (4.26, 12.45) | 9.93 (6.55, 13.34) | CG |

**Supplementary Table 2.** Details of pairwise meta-analyses

| **Comparison** | **Number of studies** | **MD** | **95%CrI** | **I2** |
| --- | --- | --- | --- | --- |
| AE vs CG | 22 | **5.86** | **(4.46, 7.26)** | 62.5 |
| BE vs CG | 8 | **4.89** | **(3.66, 6.14)** | 33.2 |
| CE vs CG | 6 | **8.51** | **(6.80, 10.23)** | 23.1 |
| HIIT vs CG | 3 | **5.07** | **(1.93, 8.21)** | 0 |
| RT vs CG | 5 | **6.7** | **(4.40, 9.00)** | 0 |

Note: Bolded numbers indicate the presence of significance. MD: mean difference. CrI: credible interval.


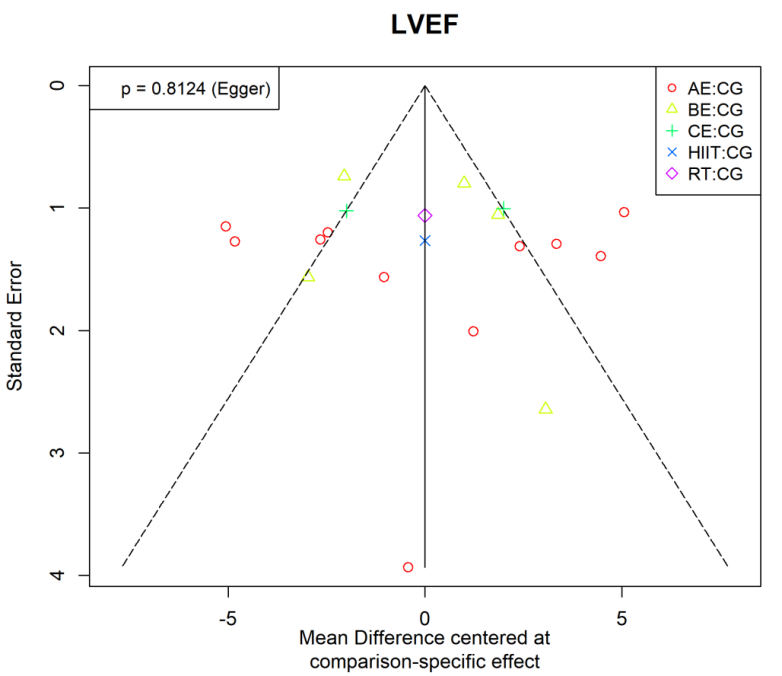


**Supplementary Figure 3.** Funnel plot.

# Appendix 6. Bayesian network meta-analysis

**Supplementary Table 3.**Model fit summaries for included studies

| **Model** | **LVEF (63 data points)** | | | |
| --- | --- | --- | --- | --- |
|  | **DIC** | **pD** | **Residual**  **Deviance** | **SD (95%CrI)** |
| FE Model | 309 | 36.1 | 272.9 | - |
| RE Model | 121.6 | 59.2 | 62.4 | 3.19 (2.36, 4.46) |
| RE UME | 122.1 | 59.4 | 62.7 | - |

Note: CrI, credible interval. Abbreviations: DIC, deviance information criterion; FE, fixed effects; pD, number of effective parameters; RE, random effects; UME, unrelated mean effects.

**Supplementary Table 4.** Consistent and UME models fit comparison.

| **Model** | **pD** | **Deviance** | **Residual deviance** | **DIC** | **SD** |
| --- | --- | --- | --- | --- | --- |
| Consistent | 60.7 | 151.020 | 63.007 | 211.6 | 4.581 |
| UME | 60.8 | 150.969 | 62.956 | 211.5 | 4.016 |

**Supplementary Table 5.** Node-split for all studies

| **Comparison** | **p.value** | **95%CrI** |
| --- | --- | --- |
| d.AE.BE | 0.503 |  |
| -> direct |  | 2.8 (-2.1, 7.7) |
| -> indirect |  | 0.82 (-2.6, 4.2) |
| -> network |  | 1.5 (-1.3, 4.2) |
| d.AE.CE | 0.86175 |  |
| -> direct |  | 0.65 (-3.4, 4.6) |
| -> indirect |  | 1.1 (-3.3, 5.6) |
| -> network |  | 0.83 (-2.2, 3.7) |
| d.AE.CG | 0.29225 |  |
| -> direct |  | -5.8 (-8.0, -3.6) |
| -> indirect |  | -3.6 (-7.2, 0.031) |
| -> network |  | -5.2 (-7.1, -3.3) |
| d.AE.RT | 0.2785 |  |
| -> direct |  | 5.6 (2.2, 9.0) |
| -> indirect |  | 1.2 (-5.9, 8.4) |
| -> network |  | 4.8 (1.7, 7.9) |
| d.BE.CE | 0.1255 |  |
| -> direct |  | -5. (-11, 1.7) |
| -> indirect |  | 0.96 (-3.1, 5) |
| -> network |  | -0.66 (-4.1, 2.9) |
| d.BE.CG | 0.09 |  |
| -> direct |  | -5.1 (-8.0, -2.1) |
| -> indirect |  | -9.6 ( -14, -5.4) |
| -> network |  | -6.6 (-9.1, -4.0) |
| d.CE.CG | 0.173 |  |
| -> direct |  | -8.5 (-13, -3.8) |
| -> indirect |  | -4.4 (-8.1, -0.58) |
| -> network |  | -6. (-9, -2.9) |
| d.CG.HIIT | 0.25425 |  |
| -> direct |  | 5.1 (-1.9, 12) |
| -> indirect |  | 10 (4.8, 16) |
| -> network |  | 8.4 (4.2, 12) |
| d.CG.RT | 0.27375 |  |
| -> direct |  | 6.8 (-0.16, 14.) |
| -> indirect |  | 11 (7.1, 15) |
| -> network |  | 10 (6.5, 13) |

# Appendix 7. Forest plot of all studies


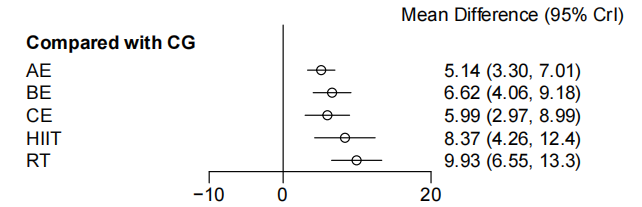


**Supplementary Figure 4.** Forest plot for all studies.

**Supplementary Table 6.** league table for sensitivity analyses

| **a.Exclude high risk of bias** | | | | | |
| --- | --- | --- | --- | --- | --- |
| AE |  |  |  |  |  |
| -1.27(-3.97, 1.45) | BE |  |  |  |  |
| -0.78(-3.54, 2.07) | 0.54(-2.85, 3.75) | CE |  |  |  |
| -3.13(-6.81, 0.61) | -1.85(-6.32, 2.61) | -2.37(-7.00, 2.16) | HIIT |  |  |
| -4.76(-7.60, -1.78) | -3.48(-7.31, 0.33) | -3.97(-7.97, -0.10) | -1.60(-6.26, 2.98) | RT |  |
| 5.45(3.43, 7.46) | 6.72(4.30, 9.14) | 6.22(3.32, 9.12) | 8.59(4.64, 12.62) | 10.20(6.94, 13.48) | CG |
| **d.Exclude interventions less than twelve weeks** | | | | | |
| AE |  |  |  |  |  |
| -0.40(-3.63, 2.93) | BE |  |  |  |  |
| -1.77(-4.82, 1.37) | -1.37(-5.49, 2.82) | CE |  |  |  |
| -3.26(-6.88, 0.57) | -2.84(-7.66, 1.91) | -1.51(-6.20, 3.33) | HIIT |  |  |
| -4.81(-7.77, -1.82) | -4.39(-8.57, -0.14) | -3.04(-7.18, 1.13) | -1.56(-6.33, 3.20) | RT |  |
| 5.00(3.01, 7.00) | 5.39(2.60, 8.24) | 6.76(3.52, 9.98) | 8.27(4.29, 12.17) | 9.76(6.49, 13.07) | CG |

# Appendix 8. Model Selection and Fitting

For our data, quadratic model shows the best fit and were therefore used in subsequent analyses.

**Supplementary Table 7.** Models fit comparison

| Model | DIC | SD | Deviance | Residual deviance | pD |
| --- | --- | --- | --- | --- | --- |
| Emax (common treatment effects) | 401.8 | NA | 365.719 | 277.706 | 36.4 |
| Restricted cubic spline (common treatment effects; 3 knots) | 378.5 | NA | 334.728 | 246.715 | 43.9 |
| Restricted cubic spline (random treatment effects; 3 knots) | 210.9 | 3.615  (2.50,5.33) | 150.556 | 62.543 | 60.5 |
| Non-parametric monotonically up (common treatment effects) | 409.9 | NA | 371.049 | 283.036 | 38.0 |
| Quadratic (2nd degree polynomial, common treatment effects) | 409.4 | NA | 368.431 | 280.418 | 41.2 |
| Quadratic (2nd degree polynomial, random treatment effects) | 209.7 | 3.684  (2.60,5.20) | 150.332 | 62.320 | 60.4 |

In addition to the model fit index, a deviation plot showing the contribution of each data point to the residuals can also help to confirm the robustness of the model selection. The contribution of each data point to the posterior mean bias should be around 1, which indicates a good model fit . Deviation plots for overall treatment effects confirm the robustness of our model selection (i.e., deviations <1.5 except for a few data points in the overall exercise and RE at 750 METs-min, which are all below a contribution of 1.5).


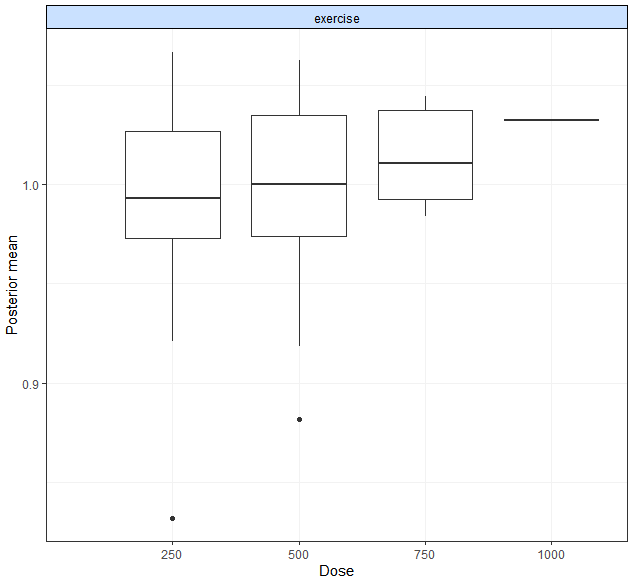


**Supplementary Figure 5.** Deviance plot at overall exercise


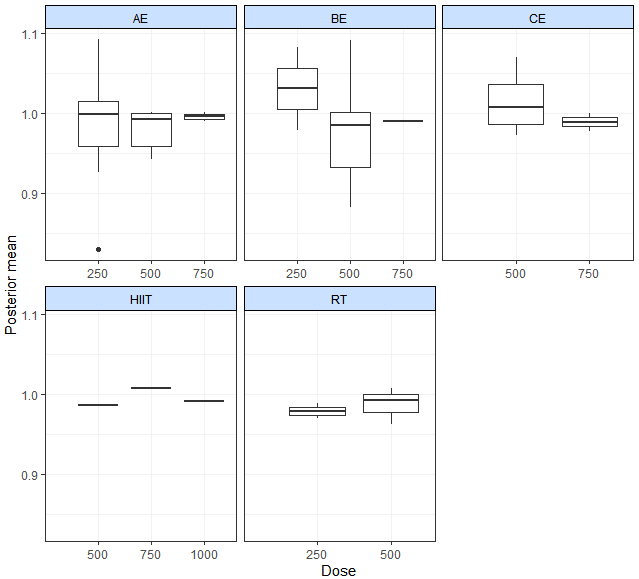


**Supplementary Figure 6.** Deviance plots at treatment-level.

In addition, we also plotted the fit further to assess the degree of fit of the model. The fit values are plotted as connecting lines, and the observations in the original dataset are plotted as points. These plots can determine if the model fits the data well for different exercises and doses of the dose-response function.


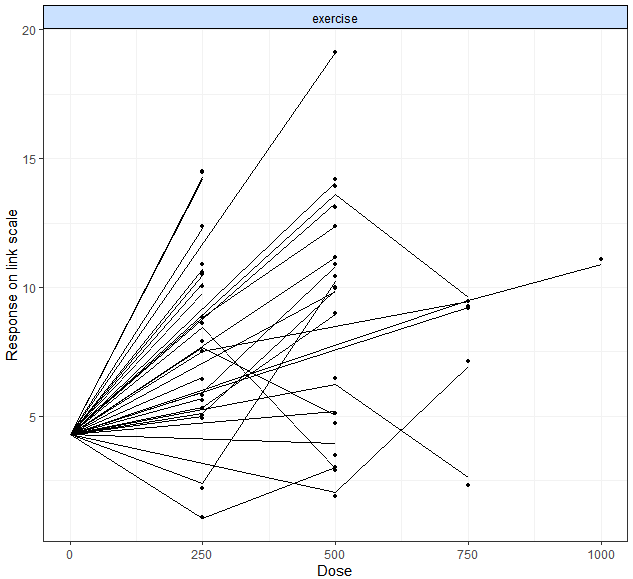


**Supplementary Figure 7.** Fit plots at overall exercise level.


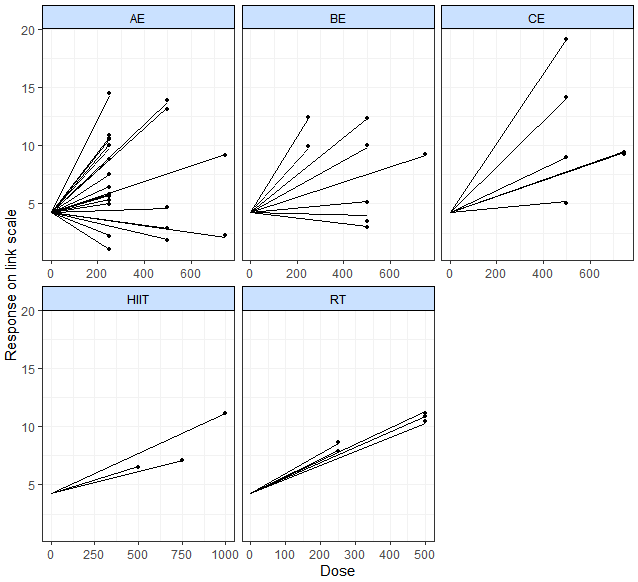


**Supplementary Figure 8.** Fit plots at agent-level. AE, continuous aerobic exercise. RE, resistance exercise. CE, combined aerobic and resistance exercise. BE, tai chi、baduanjin. HIIT, high-intensity interval training. CG, control group.

Connectivity is a key assumption in NMA that, if considered insufficient (i.e., due to lack of direct comparisons), may lead to low statistical power and misleading results . Our study assessed network connectivity at the motor and dose levels and did not find any evidence of network unconnectedness (Figure S9 and Figure S10)


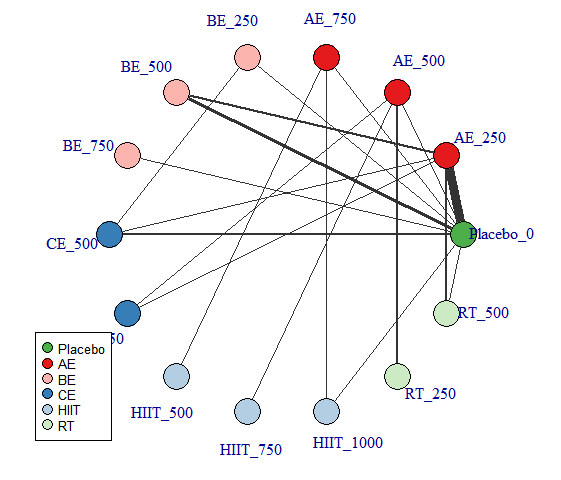


**Supplementary Figure 9.** Treatment-level network. The first value indicates the specific intervention and the second one is the corresponding dose of that intervention.


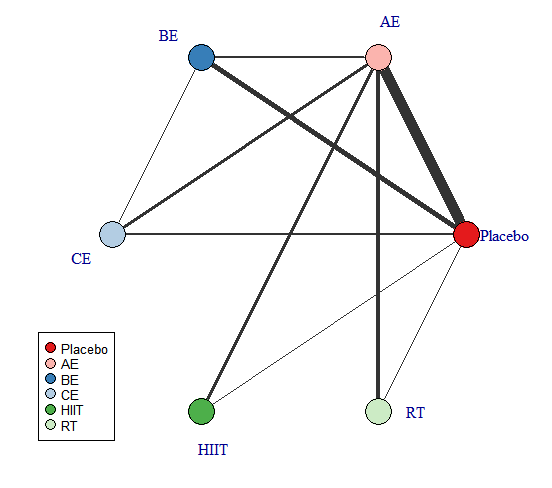


**Supplementary Figure 10.** Agent-level network. The first value indicates the specific intervention and the second one is the corresponding dose of that intervention.

**Network transitivity**

NMAs are based on the assumption of indirect/mixed comparisons, which implies that estimates of treatment effects from direct and indirect evidence are consistent but with the usual variation of meta-analyses under a random effects model . This assumption is equivalent to heterogeneity in a "standard" meta-analysis. Following a previous proposal , anomalies were assessed at a deeper network level (i.e., at the treatment level). We assessed the span by the MBNMA node-splitting method. This method splits the contribution of a specific treatment contrast into direct and indirect evidence and compares them . Similar effects indicate good span. Table S8 and Figure S11 presents the results for transitivity in this meta-analysis.

**Supplementary Table 8.** Node-splitting analysis of consistency

| **Comparison** | **p-value** | **Median** | **2.50%** | **97.50%** |
| --- | --- | --- | --- | --- |
| CE_500 vs BE_250 | 0.276 |  |  |  |
| -> direct |  | -5.019 | -14.041 | 3.733 |
| -> indirect |  | 1.725 | -2.276 | 5.669 |
| -> MBNMA |  | 0.684 | -2.981 | 4.216 |
| HIIT_500 vs AE_750 | 0.299 |  |  |  |
| -> direct |  | 4.111 | -5.199 | 13.264 |
| -> indirect |  | -3.102 | -8.09 | 1.554 |
| -> MBNMA |  | -1.758 | -6.19 | 2.592 |
| RT_250 vs AE_500 | 0.267 |  |  |  |
| -> direct |  | 4.432 | -2.127 | 10.974 |
| -> indirect |  | -1.212 | -5.039 | 2.571 |
| -> MBNMA |  | 0.264 | -3.103 | 3.757 |
| HIIT_750 vs AE_500 | 0.654 |  |  |  |
| -> direct |  | 5.145 | -3.954 | 14.32 |
| -> indirect |  | 2.372 | -3.51 | 8.012 |
| -> MBNMA |  | 3.054 | -2.007 | 7.855 |
| CE_750 vs AE_500 | 0.373 |  |  |  |
| -> direct |  | -4.59 | -14.527 | 4.825 |
| -> indirect |  | 2.248 | -3.919 | 8.006 |
| -> MBNMA |  | 0.358 | -5.043 | 5.235 |
| RT_500 vs AE_250 | 0.801 |  |  |  |
| -> direct |  | 6.58 | 0.164 | 13.426 |
| -> indirect |  | 8.412 | -0.059 | 16.058 |
| -> MBNMA |  | 7.421 | 2.377 | 12.134 |
| CE_500 vs AE_250 | 0.468 |  |  |  |
| -> direct |  | 3.77 | -5.683 | 12.659 |
| -> indirect |  | 0.586 | -2.927 | 3.903 |
| -> MBNMA |  | 0.976 | -2.459 | 4.081 |
| BE_500 vs AE_250 | 0.303 |  |  |  |
| -> direct |  | 2.818 | -3.788 | 9.469 |
| -> indirect |  | -2.871 | -7.347 | 1.585 |
| -> MBNMA |  | -2.795 | -6.371 | 0.746 |
| HIIT_1000 vs Placebo_0 | 0.251 |  |  |  |
| -> direct |  | 5.277 | -2.85 | 13.39 |
| -> indirect |  | 16.673 | 5.366 | 28.559 |
| -> MBNMA |  | 10.148 | 2.811 | 17.752 |
| BE_750 vs Placebo_0 | 0.751 |  |  |  |
| -> direct |  | 6.3 | -3.626 | 15.203 |
| -> indirect |  | 8.345 | 2.11 | 15.103 |
| -> MBNMA |  | 7.634 | 2.416 | 12.933 |
| AE_750 vs Placebo_0 | 0.484 |  |  |  |
| -> direct |  | 3.109 | -6.488 | 12.389 |
| -> indirect |  | 8.075 | 2.535 | 13.757 |
| -> MBNMA |  | 6.769 | 2.207 | 11.695 |


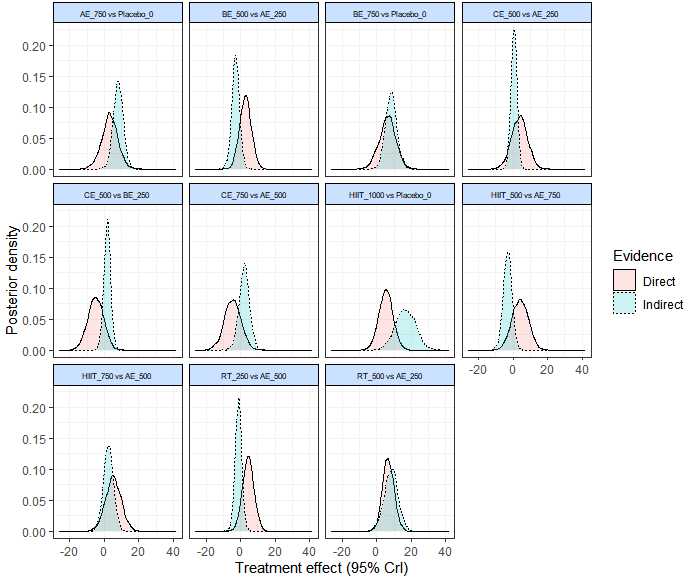


**Supplementary Figure 11.** Node-splitting analysis (density plot). The value of title is the corresponding dose of that agent. CrI, credible interval.

# Appendix 9. Predictions ranking

**Supplementary Table 9.** Predictions ranking (from best to worst)

| **Treatment** | **Mean** | **Median** | **2.50%** | **97.50%** |
| --- | --- | --- | --- | --- |
| RT_330 | 4.77 | 3 | 1 | 18 |
| RT_390 | 4.84 | 4 | 1 | 14 |
| RT_440 | 6.31 | 5 | 1 | 16 |
| RT_280 | 6.65 | 5 | 1 | 24 |
| HIIT_670 | 9.17 | 7 | 1 | 34 |
| HIIT_560 | 9.38 | 7 | 1 | 36 |
| RT_220 | 10.12 | 8 | 3 | 31 |
| RT_500 | 10.17 | 9 | 1 | 29.03 |
| HIIT_780 | 11.11 | 9 | 2 | 34 |
| HIIT_440 | 12.36 | 9 | 2 | 39 |
| RT_170 | 14.62 | 12 | 5 | 36 |
| HIIT_890 | 15.8 | 13 | 2 | 39 |
| HIIT_330 | 17.04 | 13 | 4 | 41.03 |
| BE_420 | 18.26 | 18 | 5 | 34 |
| BE_500 | 18.79 | 18 | 6 | 33 |
| BE_330 | 21.14 | 21 | 6 | 37 |
| CE_500 | 21.2 | 21 | 6 | 37 |
| AE_500 | 21.85 | 21 | 12 | 32 |
| CE_420 | 21.86 | 22 | 3.98 | 39 |
| BE_580 | 21.93 | 22 | 7 | 37 |
| AE_420 | 22.35 | 22 | 12 | 32 |
| CE_580 | 22.63 | 23 | 8 | 36 |
| AE_580 | 23.63 | 23 | 13 | 37 |
| RT_110 | 23.88 | 24 | 8 | 41 |
| HIIT_1000 | 24.34 | 24 | 1 | 46 |
| HIIT_220 | 24.74 | 24 | 7.98 | 43 |
| CE_330 | 25.16 | 26 | 5 | 41 |
| AE_330 | 25.73 | 26 | 14 | 36 |
| BE_250 | 26.14 | 27 | 10 | 40 |
| CE_670 | 26.27 | 27 | 8 | 42 |
| AE_670 | 27.35 | 28 | 11 | 44 |
| BE_670 | 27.6 | 29 | 7 | 44 |
| CE_250 | 29.74 | 32 | 9 | 43 |
| CE_750 | 30.26 | 32 | 6 | 46 |
| AE_250 | 30.57 | 31 | 18 | 39 |
| AE_750 | 31.35 | 34 | 9 | 46 |
| BE_750 | 32.77 | 36.5 | 5 | 46 |
| BE_170 | 32.95 | 34 | 18 | 43 |
| CE_170 | 35.41 | 37 | 16 | 44 |
| AE_170 | 36.22 | 37 | 26 | 42 |
| RT_56 | 37.12 | 38 | 25 | 44 |
| HIIT_110 | 37.14 | 38 | 24 | 45 |
| BE_83 | 40.81 | 42 | 31.98 | 45 |
| CE_83 | 41.86 | 43 | 31 | 45 |
| AE_83 | 42.11 | 43 | 35 | 45 |
| Placebo_0 | 45.47 | 46 | 42 | 46 |


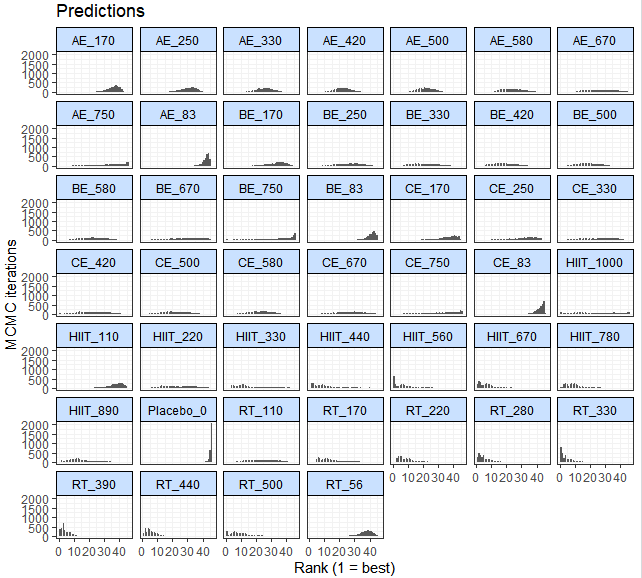


**Supplementary Figure 12.** hows the probability of each intervention to be ranked from best to worst (estimated after up to 4000iterations) Effectiveness ranking by different exercise. The number that follows the exercise intervention indicates the dose of exercise (METs-min/week). AE, continuous aerobic exercise. RT, resistance exercise. CE, combined aerobic and resistance exercise. BE, tai chi、baduanjin. HIIT, high-intensity interval training. CG, control group.

# Appendix 10. Sensitivity analysis

**Supplementary Table 10.** Model fit summaries for all studies and sensitivity analyses

| **Model** | **LVEF (63 data points)** | | | |
| --- | --- | --- | --- | --- |
|  | **DIC** | **pD** | **Residual**  **Deviance** | **SD(95%CrI)** |
|  | **All studies (63 data points)** | | | |
| FE Model | 309 | 36.1 | 272.9 | - |
| RE Model | 121.6 | 59.2 | 62.4 | 3.19 (2.36, 4.46) |
| RE UME | 122.1 | 59.4 | 62.7 | - |
|  | **Exclude high risk of bias (57 data points)** | | | |
| FE Model | 268.8 | 33.1 | 235.8 | - |
| RE Model | 109.5 | 53.2 | 56.3 | 2.99 (2.16, 4.29) |
| RE UME | 109.8 | 53.1 | 56.8 | - |
|  | **Exclude studies with interventions lasting no more than 4 weeks (55 data points)** | | | |
| FE Model | 227.4 | 32 | 195.4 | - |
| RE Model | 106.5 | 51.6 | 54.9 | 3.09 (2.20, 4.46) |
| RE UME | 107.2 | 52.2 | 54.9 | - |

***Note:*** CrI, credible interval. DIC, deviance information criterion. FE, fixed effects. pD, number of effective parameters. SD, standard deviation. RE, random effects. UME, unrelated mean effects.

**SUCRA of sensitivity analyses**

**a.** Exclude high risk of bias


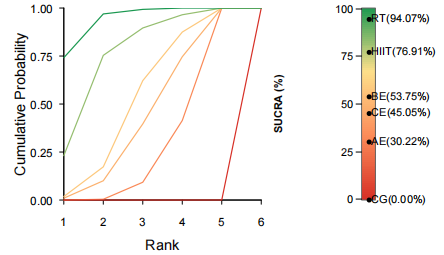


**Supplementary Figure 13-a**

b. Exclude studies with interventions lasting no more than 4 weeks


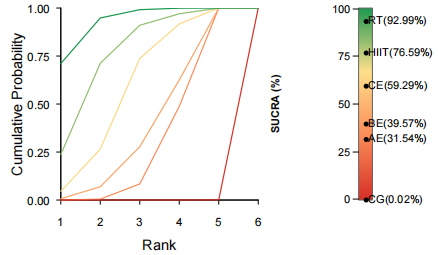


**Supplementary Figure 13-b.**

**Supplementary Table 11.** league table for sensitivity analyses

| **a.Exclude high risk of bias** | | | | | |
| --- | --- | --- | --- | --- | --- |
| AE |  |  |  |  |  |
| -1.27(-3.97, 1.45) | BE |  |  |  |  |
| -0.78(-3.54, 2.07) | 0.54(-2.85, 3.75) | CE |  |  |  |
| -3.13(-6.81, 0.61) | -1.85(-6.32, 2.61) | -2.37(-7.00, 2.16) | HIIT |  |  |
| -4.76(-7.60, -1.78) | -3.48(-7.31, 0.33) | -3.97(-7.97, -0.10) | -1.60(-6.26, 2.98) | RT |  |
| 5.45(3.43, 7.46) | 6.72(4.30, 9.14) | 6.22(3.32, 9.12) | 8.59(4.64, 12.62) | 10.20(6.94, 13.48) | CG |
| **d.Exclude interventions less than four weeks** | | | | | |
| AE |  |  |  |  |  |
| -0.40(-3.63, 2.93) | BE |  |  |  |  |
| -1.77(-4.82, 1.37) | -1.37(-5.49, 2.82) | CE |  |  |  |
| -3.26(-6.88, 0.57) | -2.84(-7.66, 1.91) | -1.51(-6.20, 3.33) | HIIT |  |  |
| -4.81(-7.77, -1.82) | -4.39(-8.57, -0.14) | -3.04(-7.18, 1.13) | -1.56(-6.33, 3.20) | RT |  |
| 5.00(3.01, 7.00) | 5.39(2.60, 8.24) | 6.76(3.52, 9.98) | 8.27(4.29, 12.17) | 9.76(6.49, 13.07) | CG |

**Supplementary Table 12.** Model fit summaries for univariate network meta-regression.

| **Covariate** | **LVEF (63 data points)** | | | | |
| --- | --- | --- | --- | --- | --- |
|  | **DIC** | **pD** | **Residual**  **Deviance** | **Shared beta**  **(Median and 95% CrI)** | **SD** |
| Unadjusted | 121.6 | 59.2 | 62.4 | - | 3.19 (2.36, 4.46) |
| Age | 121.5 | 59.1 | 62.4 | 2.22 (-0.68, 5.02) | 3.08 (2.25, 4.36) |
| week | 122 | 59.3 | 62.7 | -1.34 (-4.60, 1.81) | 3.18 (2.33, 4.50) |
| Frequency | 121.7 | 59.3 | 62.4 | 1.09 (-1.75, 3.78) | 3.22 (2.36, 4.54) |
| Publication date | 122.2 | 59.6 | 62.6 | 0.33 (-2.86, 3.46) | 3.25 (2.40, 4.56) |
| Proportion of Men | 121.9 | 59.4 | 62.5 | 0.28 (-2.88, 3.63) | 3.25 (2.39, 4.59) |
| sampleSize | 122.3 | 59.6 | 62.7 | 0.34 (-3.00, 3.49) | 3.25 (2.38, 4.56) |

***Note:*** CrI, credible interval. DIC, deviance information criterion. SD, standard deviation

**References**

1. Austin J, Williams R, Ross L, Moseley L, Hutchison S. Randomised controlled trial of cardiac rehabilitation in elderly patients with heart failure. Eur J Heart Fail. 2005;7(3):411-417. doi:10.1016/j.ejheart.2004.10.004
2. Belardinelli R, Georgiou D, Cianci G, Purcaro A. Randomized, controlled trial of long-term moderate exercise training in chronic heart failure: effects on functional capacity, quality of life, and clinical outcome. Circulation. 1999;99(9):1173-1182. doi:10.1161/01.cir.99.9.1173
3. Brubaker PH, Moore JB, Stewart KP, Wesley DJ, Kitzman DW. Endurance exercise training in older patients with heart failure: results from a randomized, controlled, single-blind trial. J Am Geriatr Soc. 2009;57(11):1982-1989. doi:10.1111/j.1532-5415.2009.02499.x
4. Chrysohoou C, Angelis A, Tsitsinakis G, et al. Cardiovascular effects of high-intensity interval aerobic training combined with strength exercise in patients with chronic heart failure. A randomized phase III clinical trial. Int J Cardiol. 2015;179:269-274. doi:10.1016/j.ijcard.2014.11.067
5. Chen YW, Wang CY, Lai YH, et al. Home-based cardiac rehabilitation improves quality of life, aerobic capacity, and readmission rates in patients with chronic heart failure. Medicine (Baltimore). 2018;97(4):e9629. doi:10.1097/MD.0000000000009629
6. Chien CL, Lee CM, Wu YW, Wu YT. Home-based exercise improves the quality of life and physical function but not the psychological status of people with chronic heart failure: a randomised trial. J Physiother. 2011;57(3):157-163. doi:10.1016/S1836-9553(11)70036-4
7. Chen XL, Jiang H, Zhong YM, et al. Clinical value of walking exercise training in patients with coronary heart disease and heart failure[J]. Chin Circ J, 2015, 30(12): 1170-1172.
8. Conraads VM, Beckers P, Vaes J, et al. Combined endurance/resistance training reduces NT-proBNP levels in patients with chronic heart failure. Eur Heart J. 2004;25(20):1797-1805. doi:10.1016/j.ehj.2004.07.022
9. Corvera-Tindel T, Doering LV, Woo MA, Khan S, Dracup K. Effects of a home walking exercise program on functional status and symptoms in heart failure. Am Heart J. 2004;147(2):339-346. doi:10.1016/j.ahj.2003.09.007
10. Dalal HM, Taylor RS, Jolly K, et al. The effects and costs of home-based rehabilitation for heart failure with reduced ejection fraction: The REACH-HF multicentre randomized controlled trial. Eur J Prev Cardiol. 2019;26(3):262-272. doi:10.1177/2047487318806358
11. Du H, Newton PJ, Budhathoki C, et al. The Home-Heart-Walk study, a self-administered walk test on perceived physical functioning, and self-care behaviour in people with stable chronic heart failure: A randomized controlled trial. Eur J Cardiovasc Nurs. 2018;17(3):235-245. doi:10.1177/1474515117729779
12. Dracup K, Evangelista LS, Hamilton MA, et al. Effects of a home-based exercise program on clinical outcomes in heart failure. Am Heart J. 2007;154(5):877-883. doi:10.1016/j.ahj.2007.07.019
13. Hambrecht R, Niebauer J, Fiehn E, et al. Physical training in patients with stable chronic heart failure: effects on cardiorespiratory fitness and ultrastructural abnormalities of leg muscles. J Am Coll Cardiol. 1995;25(6):1239-1249. doi:10.1016/0735-1097(94)00568-B
14. Hwang R, Bruning J, Morris NR, Mandrusiak A, Russell T. Home-based telerehabilitation is not inferior to a centre-based program in patients with chronic heart failure: a randomised trial. J Physiother. 2017;63(2):101-107. doi:10.1016/j.jphys.2017.02.017
15. Giannuzzi P, Temporelli PL, Corrà U, Tavazzi L; ELVD-CHF Study Group. Antiremodeling effect of long-term exercise training in patients with stable chronic heart failure: results of the Exercise in Left Ventricular Dysfunction and Chronic Heart Failure (ELVD-CHF) Trial. Circulation. 2003;108(5):554-559. doi:10.1161/01.CIR.0000081780.38477.FA
16. Karapolat H, Demir E, Bozkaya YT, et al. Comparison of hospital-based versus home-based exercise training in patients with heart failure: effects on functional capacity, quality of life, psychological symptoms, and hemodynamic parameters. Clin Res Cardiol. 2009;98(10):635-642. doi:10.1007/s00392-009-0049-6
17. Kitzman DW, Whellan DJ, Duncan P, et al. Physical Rehabilitation for Older Patients Hospitalized for Heart Failure. N Engl J Med. 2021;385(3):203-216. doi:10.1056/NEJMoa2026141
18. Kiilavuori K, Sovijärvi A, Näveri H, Ikonen T, Leinonen H. Effect of physical training on exercise capacity and gas exchange in patients with chronic heart failure. Chest. 1996;110(4):985-991. doi:10.1378/chest.110.4.985
19. Koukouvou G, Kouidi E, Iacovides A, Konstantinidou E, Kaprinis G, Deligiannis A. Quality of life, psychological and physiological changes following exercise training in patients with chronic heart failure. J Rehabil Med. 2004;36(1):36-41. doi:10.1080/11026480310015549
20. Lin BS, Xu YJ. Effect of pulmonary function rehabilitation nursing based on 5E rehabilitation model on patients with chronic pulmonary heart disease complicated with heart failure[J]. Prevent Treat Cardiovasc Dis, 2025, 15(17): 126-129.
21. Luo ZM, Li R, Zhao HY, et al. Effects of combined resistance-aerobic training on cardiac function, exercise endurance and quality of life in patients with chronic heart failure[J]. Chin Gen Pract, 2014, 17(13): 1490-1494.
22. Lundgren KM, Langlo KAR, Salvesen Ø, et al. Feasibility of telerehabilitation for heart failure patients inaccessible for outpatient rehabilitation. ESC Heart Fail. 2023;10(4):2406-2417. doi:10.1002/ehf2.14405
23. do Nascimento DM, Machado KC, Bock PM, et al. Functional training improves peak oxygen consumption and quality of life of individuals with heart failure: a randomized clinical trial. BMC Cardiovasc Disord. 2023;23(1):381. Published 2023 Jul 29. doi:10.1186/s12872-023-03404-7
24. Nagatomi Y, Ide T, Higuchi T, et al. Home-based cardiac rehabilitation using information and communication technology for heart failure patients with frailty. ESC Heart Fail. 2022;9(4):2407-2418. doi:10.1002/ehf2.13934
25. Oka RK, De Marco T, Haskell WL, et al. Impact of a home-based walking and resistance training program on quality of life in patients with heart failure. Am J Cardiol. 2000;85(3):365-369. doi:10.1016/s0002-9149(99)00748-1
26. O'Connor CM, Whellan DJ, Lee KL, et al. Efficacy and safety of exercise training in patients with chronic heart failure: HF-ACTION randomized controlled trial. JAMA. 2009;301(14):1439-1450. doi:10.1001/jama.2009.454
27. Peng X, Su Y, Hu Z, et al. Home-based telehealth exercise training program in Chinese patients with heart failure: A randomized controlled trial. Medicine (Baltimore). 2018;97(35):e12069. doi:10.1097/MD.0000000000012069
28. Piotrowicz E, Pencina MJ, Opolski G, et al. Effects of a 9-Week Hybrid Comprehensive Telerehabilitation Program on Long-term Outcomes in Patients With Heart Failure: The Telerehabilitation in Heart Failure Patients (TELEREH-HF) Randomized Clinical Trial. JAMA Cardiol. 2020;5(3):300-308. doi:10.1001/jamacardio.2019.5006
29. Piotrowicz E, Zieliński T, Bodalski R, et al. Home-based telemonitored Nordic walking training is well accepted, safe, effective and has high adherence among heart failure patients, including those with cardiovascular implantable electronic devices: a randomised controlled study. Eur J Prev Cardiol. 2015;22(11):1368-1377. doi:10.1177/2047487314551537
30. Piotrowicz E, Baranowski R, Bilinska M, et al. A new model of home-based telemonitored cardiac rehabilitation in patients with heart failure: effectiveness, quality of life, and adherence. Eur J Heart Fail. 2010;12(2):164-171. doi:10.1093/eurjhf/hfp181
31. Xueyu L, Hao Y, Shunlin X, Rongbin L, Yuan G. Effects of Low-Intensity Exercise in Older Adults With Chronic Heart Failure During the Transitional Period From Hospital to Home in China: A Randomized Controlled Trial. Res Gerontol Nurs. 2017;10(3):121-128. doi:10.3928/19404921-20170411-02
32. Tian HW, Tan X. Effect of network-platform-based family cardiac rehabilitation intervention on exercise tolerance, cardiac function and quality of life in patients with chronic heart failure[J]. J Pract Electrocardiol Clin Diagn, 2025, 34(05): 726-729. DOI:10.13308/j.issn.2097-5716.2025.05.019.
33. Vetrovsky T, Siranec M, Frybova T, et al. Lifestyle Walking Intervention for Patients With Heart Failure With Reduced Ejection Fraction: The WATCHFUL Trial. Circulation. 2024;149(3):177-188. doi:10.1161/CIRCULATIONAHA.123.067395
34. Wang Y, Pan W. Effect of Baduanjin exercise on cardiac rehabilitation in elderly patients with chronic heart failure[J]. Chin J Gerontol, 2021, 41(19): 4260-4263.
35. Che XY, Wu LL, Wang ML, et al. Effect of Taichi motor imagination therapy combined with resistance exercise on recovery of heart function and quality of life in patients with chronic heart failure[J]. Chin J Cardiovasc Rehabil Med, 2025, 34(04): 576-581.
36. Yao C, Li M. Effect of 6-minute walk test-based rehabilitation training on elderly patients with stable heart failure[J]. Contemp Med Forum, 2025, 23(31): 169-172.
37. Yeh GY, Wood MJ, Lorell BH, et al. Effects of tai chi mind-body movement therapy on functional status and exercise capacity in patients with chronic heart failure: a randomized controlled trial. Am J Med. 2004;117(8):541-548. doi:10.1016/j.amjmed.2004.04.016
38. Yeh GY, McCarthy EP, Wayne PM, et al. Tai chi exercise in patients with chronic heart failure: a randomized clinical trial. Arch Intern Med. 2011;171(8):750-757. doi:10.1001/archinternmed.2011.150
39. Yu ML, Jiang H, Li B, et al. Application of Tai Ji Quan exercise in heart rehabilitation for elderly patients with heart failure after myocardial infarction[J]. Chin J Rehabil Theory Pract, 2020, 26(08): 969-974.
40. Wang XT, Jia MJ, Liu YM. Effect of Tai Ji on heart failure with preserved ejection fraction patients: a randomized controlled trial[J]. Chin J Integr Tradit West Med, 2022, 42(08): 961-967.

Willenheimer R, Rydberg E, Cline C, et al. Effects on quality of life, symptoms and daily activity 6 months after termination of an exercise training programme in heart failure patients. Int J Cardiol. 2001;77(1):25-31. doi:10.1016/s0167-5273(00)00383-1

Zhang XE, Cheng B, Peng W. Effect of exercise training on exercise tolerance in patients with chronic heart failure[J]. Chin J Phys Med Rehabil, 2004, (02): 35-37.
